# *In vitro* and *in vivo* disease models of cardiac amyloidosis: progress, pitfalls, and potential

**DOI:** 10.1093/cvr/cvaf152

**Published:** 2025-09-01

**Authors:** Jiabin Qin, Zeping Qiu, Yingze Fan, Qipeng Xiong, Zhiyong Lei, Jin Wei, Pim van der Harst, Monique C Minnema, Joost P G Sluijter, Alain van Mil, Marish I F J Oerlemans

**Affiliations:** Department of Cardiology, University Medical Center Utrecht, Heidelberglaan 100, 3508 GA, Utrecht, The Netherlands; Laboratory of Experimental Cardiology, Regenerative Medicine Center Utrecht, Circulatory Health Research Center, University Utrecht, University Medical Center Utrecht, Uppsalalaan 8, 3584 CT, Utrecht, The Netherlands; Department of Cardiovascular Medicine, Ruijin Hospital, Shanghai Jiao Tong University School of Medicine, 197 Ruijin 2nd Road, Shanghai 200025, People’s Republic of China; Institute of Cardiovascular Diseases, Shanghai Jiao Tong University School of Medicine, Shanghai, People’s Republic of China; Department of Cardiovascular Medicine, Ruijin Hospital, Shanghai Jiao Tong University School of Medicine, 197 Ruijin 2nd Road, Shanghai 200025, People’s Republic of China; Institute of Cardiovascular Diseases, Shanghai Jiao Tong University School of Medicine, Shanghai, People’s Republic of China; Department of Cardiology, University Medical Center Utrecht, Heidelberglaan 100, 3508 GA, Utrecht, The Netherlands; Laboratory of Experimental Cardiology, Regenerative Medicine Center Utrecht, Circulatory Health Research Center, University Utrecht, University Medical Center Utrecht, Uppsalalaan 8, 3584 CT, Utrecht, The Netherlands; CDL Research, University Medical Center Utrecht, Utrecht, The Netherlands; Department of Cardiovascular Medicine, Ruijin Hospital, Shanghai Jiao Tong University School of Medicine, 197 Ruijin 2nd Road, Shanghai 200025, People’s Republic of China; Institute of Cardiovascular Diseases, Shanghai Jiao Tong University School of Medicine, Shanghai, People’s Republic of China; Department of Cardiology, University Medical Center Utrecht, Heidelberglaan 100, 3508 GA, Utrecht, The Netherlands; Department of Hematology, University Medical Center Utrecht, University Utrecht, Utrecht, The Netherlands; Department of Cardiology, University Medical Center Utrecht, Heidelberglaan 100, 3508 GA, Utrecht, The Netherlands; Laboratory of Experimental Cardiology, Regenerative Medicine Center Utrecht, Circulatory Health Research Center, University Utrecht, University Medical Center Utrecht, Uppsalalaan 8, 3584 CT, Utrecht, The Netherlands; Department of Cardiology, University Medical Center Utrecht, Heidelberglaan 100, 3508 GA, Utrecht, The Netherlands; Laboratory of Experimental Cardiology, Regenerative Medicine Center Utrecht, Circulatory Health Research Center, University Utrecht, University Medical Center Utrecht, Uppsalalaan 8, 3584 CT, Utrecht, The Netherlands; Department of Cardiology, University Medical Center Utrecht, Heidelberglaan 100, 3508 GA, Utrecht, The Netherlands; Laboratory of Experimental Cardiology, Regenerative Medicine Center Utrecht, Circulatory Health Research Center, University Utrecht, University Medical Center Utrecht, Uppsalalaan 8, 3584 CT, Utrecht, The Netherlands; Member of the European Reference Network for Rare, Low Prevalence and Complex Diseases of the Heart: ERN GUARD-Heart’ (ERN GUARDHEART); Transplantation Center University Medical Center Utrecht, Heidelberglaan 100, 3508 GA, Utrecht, The Netherlands

**Keywords:** Light chain, Transthyretin, Amyloid fibrils, *C. elegans*, Zebrafish, Transgenic mouse, iPSC, Proteolysis, ER stress

## Abstract

Amyloid light chain (AL) and transthyretin amyloidosis (ATTR)-induced cardiomyopathy are life-threatening protein misfolding disorders characterized by amyloid fibril deposition in the heart, which significantly impairs cardiac function. The lack of representative disease models has impeded progress in understanding the underlying mechanisms and hindered the discovery and development of specific biomarkers and effective therapies. To address this, researchers have developed various cell and animal models to recapitulate these diseases. In AL amyloidosis, cell and mouse models have highlighted the toxic effects of both soluble light chains (LCs) and LC-derived amyloid fibrils, such as lysosomal dysfunction, endoplasmic reticulum stress, and oxidative stress. Transgenic mouse models, particularly those without the mouse heavy chain and with amyloid seeds addition, have successfully replicated systemic AL amyloidosis, with clear effects on the heart. For ATTR amyloidosis, acid-induced transthyretin (TTR) fibrils induce cellular dysfunction, such as increased intracellular reactive oxygen species (ROS) level, disorganized sarcomere, and prolonged calcium handling in 2D cell models. Transgenic mouse models expressing human WT or variant TTR have offered insights into the development of amyloid cardiomyopathy, but challenges persist in fully replicating the human phenotype. This review offers a comprehensive overview of the significant advancements, challenges, and future perspectives in the development of various cell and animal models for studying AL and ATTR amyloidosis-induced cardiomyopathy, thereby providing valuable insights into disease pathophysiology, early accurate biomarkers identification, and development of novel therapies.

## 1. Introduction

Amyloidosis is a protein folding disorder that arises from extracellular fibrillar protein aggregates (i.e. amyloid fibrils) that are insoluble and resistant to proteolysis,^1^ resulting in tissue damage and organ dysfunction. Amyloid fibrils are formed by the parallel stacking and twisting of protofilaments, which themselves consist of layers of proteins in a β-sheet structure, bound together via their side chains.^2^ The classification of amyloidosis is based on the amyloidogenic precursor protein, which affects the patterns of organ deposition, the natural course of the disease, and the most appropriate treatment approach. So far, 42 proteins have been identified as being amyloidogenic in humans.^3^ Cardiac amyloidosis primarily involves the misfolding and aggregation of immunoglobulin light chains (LCs) or transthyretin (TTR) within the heart. Cardiac amyloid deposition stiffens and thickens the heart muscle, impairing ventricular relaxation and expansion. This impaired relaxation and reduced elasticity lead to diastolic dysfunction, a condition where the heart struggles to fill with blood effectively. As a result, patients often develop heart failure with preserved ejection fraction. Also, amyloid deposition disrupts the electrical conduction system of the heart, leading to a variety of arrhythmias. Over time, the relentless progression of amyloid deposition culminates in amyloid cardiomyopathy, which has a poor prognosis if left untreated.^4,5^ Early diagnosis and proper treatment are crucial to have better outcome for patients, but the non-specific symptoms and lack of specific biomarkers often result in delayed diagnosis or misdiagnosis.^6^ Since the stage of the disease at diagnosis significantly impacts patients’ survival, early and accurate diagnosis and subsequent treatment are essential to effectively impede further cardiac damage and reduce mortality.^7,8^

Significant progress has been made in the past decades in understanding the intricacies of cardiac amyloidosis by developing valuable animal and cell models that provide crucial insights into the disease. These models pave the way for a better understanding of the disease pathophysiology and facilitate the development of more effective diagnostic tools and treatments. In this review, we presented a comprehensive overview of the available animal and cell models. Next, we highlighted valuable insights gained from these research models and discussed future perspectives to improve the existing models, enabling the development of better predictive models and an increased understanding of the disease to facilitate accurate diagnosis and effective treatment.

## 2. Amyloid light chain amyloidosis pathogenesis

Amyloid light chain (AL) amyloidosis, the most prevalent systemic amyloidosis, is caused by clonal plasma cells in the bone marrow overproducing immunoglobulin lambda (λ), or less commonly, kappa (κ) LCs.^9^ One of the challenges that AL amyloidosis presents is the heterogenicity with a unique protein sequence in each patient, arising from the selection of a specific germline gene and the accumulation of somatic mutations during the immune response.^10^ These secreted amyloidogenic-free LCs are prone to misfolding, fibril formation and deposition in the heart, leading to characteristic myocardial alteration and dysfunction.^11^

### 2.1 AL fibril formation

The typical *in vitro* process of AL amyloid fibril formation has been extensively studied,^12,13^ demonstrating a process that starts with a lag phase where misfolded LCs gradually aggregate into non-fibrillar oligomers, protofilaments, and mature fibrils. However, the mechanisms that cause the initial misfolding of LCs remain unclear, with the potential involvement of LC gene heterogenicity and post-translational proteolysis.

### 2.2 Mutations in the LC gene

LCs, as part of monoclonal immunoglobulins, are ∼22 kDa proteins composed of two β-sheet rich domains: the N-terminal variable domain (V_L_) and the C-terminal constant domain (C_L_).^14^ Particularly, V_L_, accommodating hypervariable complementarity-determining regions (CDR), displays a high number of non-conservative mutations compared with its germline counterpart.^15^ Mutations in these areas contribute to extensive sequence variability and thermodynamic destabilization, rendering the LCs more susceptible to misfolding and fibril formation.^16–26^ Cryo-EM analysis has shown significant structural rearrangements of the V_L_ in AL fibrils from patients’ amyloid deposits, where the internal disulfide bond remains intact but the hydrophobic core residues become exposed on the surface.^27,28^ Also, chromosomal alterations, like the 14q32 immunoglobulin heavy chain translocations, are frequently observed in amyloidogenic plasma cells, and are frequently associated with an increased free LC level in AL amyloidosis patients.^29^ Together with the high level of free LCs in the serum and other body fluids of AL patients, mutations in LCs provide a foundation for subsequent steps for amyloid fibril formation.^30–32^

### 2.3 Proteolytic cleavage in the LC protein

In addition to gene and protein mutations in LCs, post-translational truncation by proteolysis has been proposed as a critical factor in LC fibril formation. While the precise role of proteolysis in AL amyloidosis remains to be fully elucidated, numerous studies on AL patients’ biopsies have shown C-terminally truncated LCs and V_L_ domains are the primary components of amyloid deposits.^19,33–35^  *In vitro* studies with bacterial-generated recombinant LCs further support the amyloidogenic potential of the V_L_ domain and the potential stabilizing effects of the C_L_ domain,^36–38^ implying the importance of proteolytic cleavage in fibril formation. In contrast, mass spectrometry analysis of amyloid-laden tissue has also detected full-length LCs or even C_L_ domains,^35,39,40^ suggesting a more complex picture where amyloid fibrils in AL amyloidosis may consist of a mixture of variable, variable and constant domain fragments, and full-length LCs.

## 3. ATTR amyloidosis pathogenesis

ATTR amyloidosis is categorized into two types based on the TTR gene sequence: wild-type ATTR (ATTRwt), and hereditary or variant ATTR (ATTRv). ATTRwt, defined with a normal TTR genetic sequence, mainly affects the elderly population with cardiomyopathic complications and has been recognized as an overlooked cause of heart failure.^29,41^ Although the exact mechanism of WT TTR destabilization and aggregation remains unclear, the ageing process appears to play a significant role.^42,43^ ATTRv results from specific point mutations in the TTR gene, leading to amino acid substitution and consequent instability.^44^ Unlike ATTRwt, the variant type manifests at an earlier age and can affect other organs like the nervous system.^45^ To date, over 120 mutant single-nucleotide polymorphism variants have been identified within the TTR gene. The most prevalent types are Val30Met (common in Portugal, Japan, and Sweden) and Val122Ile (in African Americans), where the amino acid valine is, respectively, replaced by either methionine or isoleucine at positions 30 or 122 of the TTR amino acid sequence. The Val30Met ATTRv predominantly manifests with polyneuropathy and may progress to cardiomyopathy later in the disease course, causing heart conduction disorder and heart failure.^46,47^ In contrast, the Val122Ile ATTRv is associated with a higher frequency of congestive heart failure,^48^ and is present in 3.5% of the African-American population.^49^

### 3.1 TTR protein and gene

TTR, formerly named pre-albumin, is a homotetrameric 55 kDa non-glycosylated protein composed of four identical subunits. It is synthesized mainly in the liver and abundantly present in human plasma, functioning as a transport protein for thyroid hormone thyroxine (T4) and retinol-binding protein 4 (RBP4).^50^ Each TTR monomer is composed of 127 amino acids with eight β-sheet chains. Monomeric TTR forms a robust, stable dimeric structure primarily through extensive hydrogen bonding. These dimers then weakly associate with each other to construct the final tetrameric structure via hydrophobic interactions.^50,51^

### 3.2 TTR fibril formation

The underlying mechanisms leading to TTR fibril formation are not completely understood, yet several proposed mechanisms likely contribute to this complex process. The tetrameric TTR dissociation has been considered as the rate-limiting step in TTR fibril formation *in vitro*, and presumably *in vivo*.^44,52^ These released monomers, upon partial unfolding, initiate a downhill polymerization process, sequentially forming oligomers, soluble aggregates, insoluble aggregates, and ultimately amyloid fibrils.^53,54^ This hypothesis has been further supported by *in vitro* studies using engineered monomeric TTR in the presence of amyloid seeds from ATTR patients.^55,56^ However, the acid-induced amorphous TTR aggregation is structurally and possibly functionally distinct from the amyloid fibrils that form naturally within the body.^45^

Amyloid seeds, essentially fragments of pre-existing mature fibrils from patients, act as a nucleus or template to trigger self-propagating amyloidogenic processes upon encountering amyloid precursor protein.^57^ Saelices *et al*.^56,58^ demonstrated that ATTR amyloid seeds can trigger the fibril formation with native-like structures when incubating with monomeric TTR under physiological conditions (pH = 7.4). This seeding effect was not observed with the tetrameric form of TTR. This critical observation highlights the importance of amyloid seeds in fibril formation and may explain why cardiac and neurological symptoms can sometimes progress in patients even after undergoing orthotopic liver transplantation to suppress primary variant TTR production. In these clinical cases, existing amyloid deposits may continue to act as seeds, promoting the aggregation of misfolded TTR.^59,60^

In addition to TTR dissociation, two distinct patterns of TTR amyloid deposits have been identified in patients, either a mixture of full-length and cleaved fragmented TTR (referred to as Type-A), or only full-length TTR (referred to as Type B). Type-A fibrils are predominantly composed of TTR fragments, with the 49–127 fragment being most prevalent in tissue biopsies of cardiac deposits, while full-length TTR is minimally present. In contrast, type B fibrils are mainly comprised of full-length TTR.^61–64^ These findings indicate alternative, proteolysis-involved pathways for TTR fibrillogenesis. A similar amyloidogenic process has been recapitulated under trypsin-like protease digestion, where Ser52Pro variant TTR exhibited a selective proteolytic cleavage at residue 48, resulting in the release of the potently amyloidogenic residue 49–127 fragment and rapid fibril formation.^65–68^

Despite advances in our understanding of the fibril formation process, the precise mechanisms initiating amyloid deposition in the heart remain elusive. Large TTR aggregates have been identified in the plasma of patients with cardiac ATTR amyloidosis.^69,70^ These aggregates indicate the presence of high molecular weight oligomers that may drive the formation of ATTR aggregates prior to deposition. However, further investigation is needed to understand how amyloid fibril deposition occurs in the heart at later stages of the disease.

## 4. AL cardiomyopathy research models

### 4.1 AL *in vitro* cell models

#### 4.1.1 Cardiomyocyte cell models

A key aim in AL amyloidosis research is identifying the most toxic species involved in amyloid fibril formation. Experiments using soluble free LCs isolated from AL amyloidosis patients have revealed that, in addition to the physical disruption caused by AL fibril deposition, circulating free LCs can also have a direct, detrimental impact on heart function (*Table 1*).

**Table 1:** Summary of AL cardiomyocyte cell models

Amyloid materials	Cell models	Key phenotypes	References
LCs isolated from AL cardiomyopathy patients	Langendorff-perfused isolated mouse hearts	Impaired ventricular relaxation Preserved contractile function	^71^
	Adult rat ventricular cardiomyocytes	Increased ROS level Impaired lysosomal function Impaired autophagic flux Impaired cardiomyocyte contractility and relaxation	^72–75^
Fibrils induced from human myeloma sera LCs	Rat cardiomyocytes H9c2 cells	Cell surface attached fibrils Reduced cell viability Increased ROS level Excessive autophagy	^76^
Recombinant full-length LC or V-aLC	Mouse HL-1 atrial cardiomyocytes AC ventricular cardiomyocyte lines	Reduced cell viability Increased caspase activity Cellular internalization of full-length LCs and V-aLCs Up-regulation of myogenesis hallmark pathway target genes	^77–80^
Recombinant full-length LC or V-aLC-induced fibrils	AC ventricular cardiomyocyte lines	Cell surface–attached fibrils Impaired metabolic activity Increased ROS level Up-regulation of cytokine and chemokine transcripts Protected by co-cultured MSCs	^81–83^

**Figure 1:**
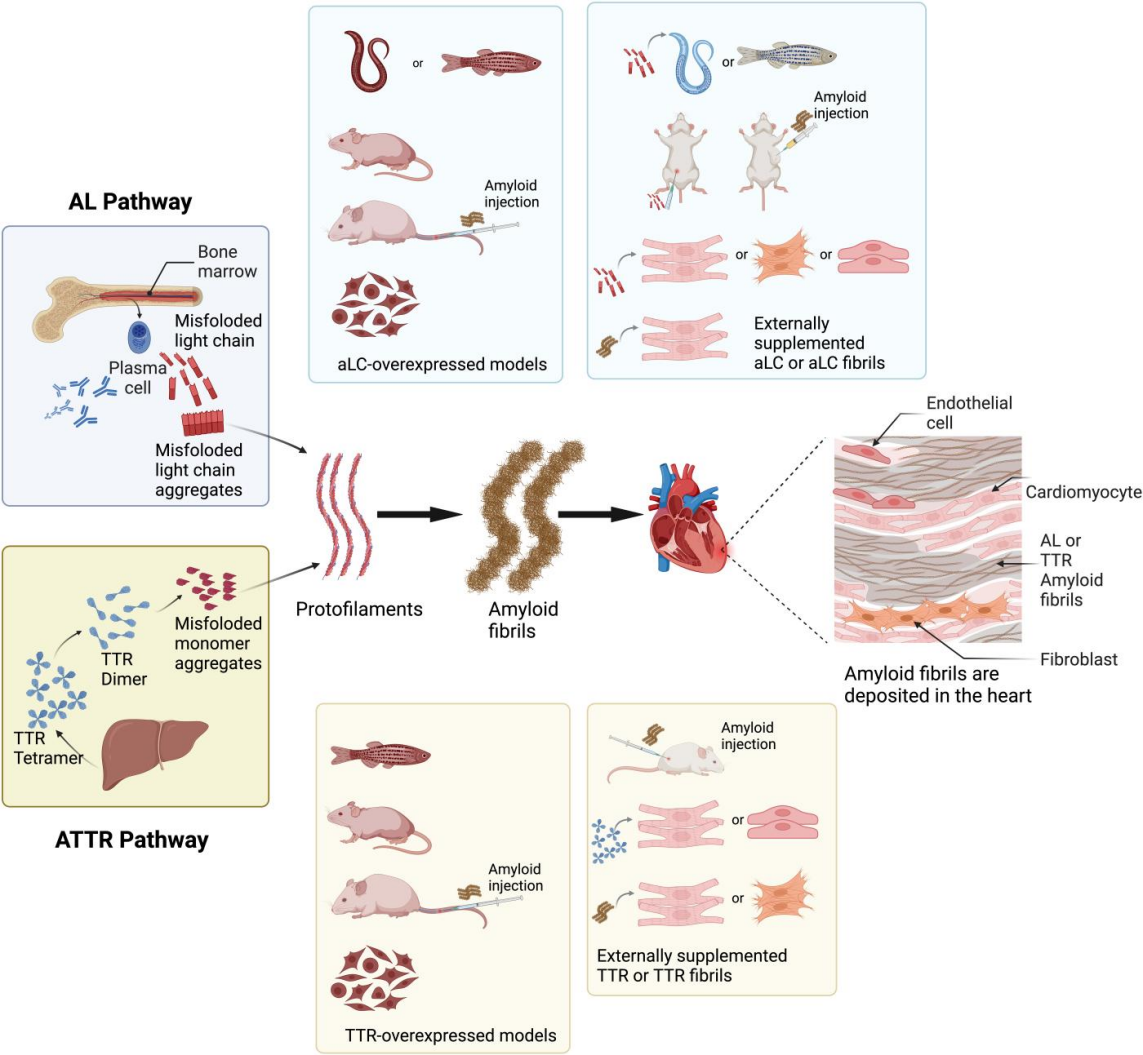
Overview of *in vitro* and *in vivo* research models for AL and ATTR cardiac amyloidosis. Cardiac amyloidosis is primarily caused by the misfolding of LCs, produced by plasma cells in the bone marrow, or transthyretin (TTR), primarily produced in the liver. Both pathways result in the formation of amyloid fibrils that deposit in the heart, leading to cardiac dysfunction. Various *in vitro* and *in vivo* models were used to study AL and ATTR amyloidosis, including cell and animal models with LC and TTR genetic modifications, and models with externally supplemented LC, TTR, and derived fibrils. Created with Biorender.com.

In 2001, Liao *et al*.^71^ firstly demonstrated the proteotoxic effects of amyloidogenic LCs (aLCs) from AL amyloidosis patients with severe cardiac involvement, like heart failure (cardiac-aLCs). Various types of LCs, including aLCs from cardiac involvement patients with or without heart failure, no cardiac involvement, or from patients without amyloidosis, were infused into isolated, *ex vivo* contracting mouse hearts (*Figure 1*). The study showed that only the cardiac-aLCs from heart failure patients impaired ventricular relaxation while preserving contractile function, like the diastolic dysfunction phenotype observed in AL cardiomyopathy patients. The other types of LCs did not influence *ex vivo* heart performance. Subsequent studies have supported these initial findings, showing that administering cardiac-aLC at physiological concentration (20 mg/L) to isolated adult rat ventricular cardiomyocytes (NRVCMs) specifically increased the intracellular ROS level, impaired lysosomal function, autophagic flux, and cardiomyocyte contractility and relaxation.^72,73^ Additionally, p38 mitogen-activated protein kinase (MAPK) and mammalian stanniocalcin1 (STC1) were identified as key mediators in cardiac-aLC-induced apoptosis. These proteins were up-regulated in patient tissue samples, an AL zebrafish model, and NRVCMs when exposed to cardiac-aLCs. Importantly, inhibiting p38 MAPK activity or knocking down STC1 significantly protected against aLC-induced cell death and improved cellular function, highlighting the critical role of p38 MAPK and STC1 in the pathogenesis of AL cardiomyopathy.^74,75^

Sikkink and Ramirez-Alvarado^77,78^ pioneered the investigation into the cytotoxicity and internalization of recombinant aLC variable domain (V_L_-aLC) using mouse HL-1 atrial cardiomyocytes and immortalized human AC ventricular cardiomyocyte lines. They found that V_L_-aLC, when supplemented at pathophysiological concentrations (exceeding 200 mg/L), significantly reduced cell viability and induced apoptosis with up-regulated caspase activity over time, relative to the corresponding germline protein. Furthermore, internalized V_L_-aLC in mouse HL-1 cardiomyocytes were found to co-localize within lysosome compartments through macropinocytosis, leading to lysosomal expansion.^78,79^ RNAseq data revealed that AC16 cardiomyocytes exposed to a physiological concentration of free LCs (∼20 mg/L) exhibited profound endoplasmic reticulum (ER) stress and up-regulation of genes associated with myogenesis, potentially as a protective mechanism in response to stress.^80^

While aLC toxicity has been widely studied, recent findings suggest that the AL fibrils themselves may directly cause damage to the heart as well. AL fibrils generated from λ LCs can reduce the viability of embryonic rat myocardium-derived H9c2 cells. This cytotoxic effect is attributed to the attachment of LC fibrils to the cell surface, triggering oxidative stress and excessive autophagy via the Foxo3a/Beclin-1 pathway.^76^ Also, V_L_-aLC-induced fibrils, generated at pH 2.0 or through agitation, showed cytotoxicity in AC10 and AC16 cardiomyocytes. Similarly, these fibrils were observed to attach to the cell surface to initiate a seeding effect^78^ and severely impaired metabolic activity and cell growth in a dose-dependent manner.^81^ These effects could be reversed by co-culturing adipose-derived mesenchymal stromal cells (aMSC) with AC16 cardiomyocytes, where aMSC rescued the AC16 cardiomyocytes from fibril-induced growth arrest through contact-dependent mechanisms and potential secreted factors.^82^ Transcriptomic data for this co-culture model revealed complement pathway activation and up-regulation of cytokine and chemokine expression in cardiomyocytes, suggesting that AL fibrils stimulate an innate immune response on these cells.^83^

#### 4.1.2 Fibroblast cell models

Cardiac fibroblasts, which comprise a significant percentage (10–25%) of the cellular population in the heart muscle, are essential for maintaining cardiac structure by regulating the organization and turnover of the elastic extracellular matrix (ECM).^84^ The interaction between amyloid fibrils and the ECM could contribute to the stiffening of the cardiac tissue. Therefore, cardiac fibroblasts are likely to play an important role in the overall dysfunction associated with amyloid cardiomyopathy.

Exposure of human cardiac fibroblasts (hCFs) to cardiac-aLCs from AL cardiomyopathy patients resulted in reduced cell viability, accompanied by activation of apoptotic pathways and oxidative stress (*Table 2*). Extensive proteome remodelling was also observed, which affected essential cellular processes in hCFs, including cytoskeletal organization, protein synthesis and quality control, mitochondrial activity, metabolism, signal transduction, and molecular trafficking.^85^ This proteotoxicity on hCFs could be mitigated by engineering the amino acid sequence of the highly cardiotoxic aLCs to reduce the dynamics of its native state. Although the cardiac-aLCs and the engineered aLCs shared similar crystal structures, cardiac-aLCs displayed a more dynamic native state with higher proteotoxicity, suggesting a strong correlation between the conformational properties of the native protein folding and the proteotoxic potential of cardiac-aLCs.^86^

**Table 2:** Summary of AL other cell models

Amyloid materials	Cell models	Key phenotypes	References
LCs isolated from AL cardiomyopathy patients	Primary human cardiac fibroblasts	Reduced viability Activated apoptotic pathways Increased ROS level Proteome remodelling Cellular internalization of LCs into mitochondria Mitochondria morphology change	^85–87^
	Primary rat cardiac fibroblasts	Cellular internalization of LCs into lysosomes Proteoglycan expression and localization modification	^79,88^
	Human adipose arterioles and coronary arterioles	Increased apoptosis Reduced nitrosative stress Increased oxidative stress	^89–93^
Cell-expressed aLCs	ALMC-1 and ALMC-2 cell lines Mouse NIH3T3 fibroblasts B lymphocyte cell lines	Secreted folded and full-length LCs Platform for siRNA screening	^94–97^
	Human THP-1 monocytes HEK cells	Extracellular aLC aggregates ingested into endosome Attenuated secretion of aLC	^98,99^

aLCs have also been reported to be encapsulated by primary rat cardiac fibroblasts through macropinocytosis as shown by their presence in lysosomes, resulting in modification of proteoglycan expression and localization.^88,100^ In 2015, Lavatelli *et al*. applied a functional proteomic approach, based on direct and inverse coimmunoprecipitation and mass spectrometry, to explore the molecular interactions between aLC and hCFs components. In addition to discovering the presence of aLC in endolysosomal compartments, they observed wider cristae in mitochondria and co-localization of cardiac-aLCs with the mitochondria protein OPA1, which resides in the inner mitochondrial membrane and functions in regulating mitochondrial fission–fusion equilibrium. Importantly, these colocalizations did not occur in the LCs from non-amyloidogenic patients. This unique finding for cardiac-aLCs highlights the potential for direct interactions between toxic aLCs and mitochondria, which could contribute to the development of mitochondrial dysfunction and ultimately, cardiac damage in AL amyloidosis (*Figure 1*).^87^

#### 4.1.3 Endothelial cell models

Studies have shown that early endothelial microcirculatory dysfunction in AL amyloidosis patients is associated with neuropathy.^101^ Further research has indicated a correlation between the presence of LCs and histological evidence of myocardial ischaemia in most AL amyloidosis patients,^102^ and LC amyloid infiltration within epicardial coronary arteries is prevalent in almost all analysed AL amyloidosis cases.^103^ Collectively, these observations suggest that microvascular dysfunction is clearly involved in the disease progression of AL amyloidosis. However, the pathophysiological mechanisms underlying endothelial dysfunction and its role in the overall AL disease process require further study.

Migrino *et al*. were the first to demonstrate aLC toxicity on the microvasculature. They found that acute aLC exposure induced endothelial dysfunction in both human adipose arterioles and coronary arterioles. In addition, aLC-induced apoptosis and necrosis through oxidative and nitrosative stress was shown in human coronary artery endothelial cells, which could be reverted by treatment with superoxide dismutase.^89,90^ Further research identified a constitutively expressed glycoprotein; clusterin, as a potential protective glycoprotein against aLC-induced endothelial dysfunction. Clusterin may bind to various proteins in a partially unfolded or stressed state, thereby preventing their aggregation and subsequent precipitation by forming large, soluble complexes.^104^ To enhance its protective effects, specific nanoliposomes, artificial phospholipid vesicles containing clusterin, were developed.^105^ Co-treatment with aLCs and nanoliposomes reversed endothelial dysfunction and cell death (*Table 2*).^91–93^ Beyond AL amyloidosis, these nanoliposomes have also shown promise in reversing endothelial dysfunction caused by other amyloid proteins, such as β-amyloid.^106–108^ This innovative approach using clusterin-loaded nanoliposomes offers a potential new class of therapeutic agents for mitigating tissue injury in AL amyloidosis.

#### 4.1.4 Immune cell models

Chemotherapy that effectively reduces free LC levels in the blood has been shown to improve cardiac biomarker (e.g. N-terminal pro-B-type natriuretic peptide (NT-proBNP)) levels and patient survival, even without significantly reducing amyloid deposits in the heart. This suggests that blocking LC production from clonal plasma cells is a promising avenue for the development of new and effective treatments for AL amyloidosis.^109^ One approach to blocking LC production involves using RNA interference (RNAi). A pool of short-interfering RNA (siRNA) targeting the constant region of λ aLC has been shown to rapidly and substantially reduce aLC production and secretion,^94^ regardless of the unique variable region gene sequences. This study utilized ALMC cell lines, which were derived from plasma cells isolated from an AL amyloidosis patient with cardiac involvement before and after stem cell transplant, respectively.^95^ Both cell lines secreted identical, fully folded, and full-length λ LCs, despite some genetic differences were observed. The efficacy of siRNA in reducing LC production was also observed in aLC-overexpressed mouse NIH3T3 cells, four human cell lines (ARH-77, NCI-H929, IM-9, and JJN-3) that produce κ LCs, and 20 specimens of CD138-selected marrow plasma cells from patients with κ plasma cell diseases (*Table 2*).^96,97^ However, in siRNA-treated plasma cells that secrete complete immunoglobulin, increased unpaired immunoglobulin heavy chains led to the up-regulation of ER stress signalling and apoptosis. This was mediated through the unfolded protein response (UPR) and endoplasmic-reticulum-associated protein degradation (ERAD) pathways. This potential risk to normal plasma cells needs careful consideration in future clinical trials. Additionally, comparing this approach's efficacy to current chemotherapy treatments for free LCs reduction is necessary, as both methods target normal plasma cells.^110^

Monoclonal antibodies (mAbs) are emerging as potential tools for both imaging and therapy in amyloidosis.^111^ The murine-derived mAb 2A4, a variant of NEOD001, possesses conformation-specific binding affinity for insoluble aLC aggregates extracted from patients tissue and synthetic fibrils.^112^ Also, macrophages were found within amyloid deposits and adjacent area of 2A4-treated mice. To understand this phenomenon, Renz *et al*.^98^ incubated human monocyte THP-1 cells with mAb 2A4 and insoluble AL aggregates from heart extracts or AL fibrils derived from ALMC cell lines. They observed that the monocytes effectively ingested aLC aggregates into acidic compartments, like the phagocytic endosome, accompanied with a reduction in extracellular aggregates. This suggests that 2A4 can promote phagocytosis of aLC aggregates via macrophages. These findings highlight the potential of mAbs, specifically 2A4, in targeting and clearing amyloid deposits. Despite being safe and tolerable in patients with AL amyloidosis in a Phase I/II study, the humanized IgG1 mAb Birtamimab (NEOD001) failed to meet the primary endpoint of cardiac response after 12 months of follow-up and the planned Phase III trial (VITAL) was ceased prematurely.^113,114^ Another monoclonal IgG1 antibody anselamimab (CAEL-101), a chimeric form of murine mAb 11-1F4, has shown promising results in a Phase Ia/b study. Further investigation with a larger patient population is ongoing to evaluate the drug efficacy.^114,115^

### 4.2 AL *in vivo* animal models

#### 4.2.1 *Caenorhabditis elegans* models

The nematode *Caenorhabditis elegans*, known for its rhythmically pumping pharynx analogous to the vertebrate heart, has become a valuable model in biomedical research, including in the field of cardiology.^116^ Merlini's group developed the first transgenic *C. elegans* model expressing a human λ aLC derived from an AL cardiomyopathy patient (referred to as MNH) as well as control strains expressing non-aLCs from an MM patient (referred to as MNM) or an empty vector (referred to as MNV) (*Figure 1*).^117^ The MNH *C. elegans* produced a soluble, dimeric protein in the body-wall muscle, resembling those found in AL amyloidosis patients. Although the amount of amyloid deposits was minimal, the secreted cardiac-aLCs caused specific damage to the pharynx, leading to reduced contraction frequency, increased superoxide production, and mitochondrial ultrastructural damage, which were not seen in the control groups (MNM and MNV *C. elegans*). These findings, including the pharyngeal defect, increased mitochondrial ROS production and marked mitochondrial structural damage, align with previous studies where the same cardiac-aLCs extracted from patients’ body fluid were administered to WT *C. elegans*.^118,119^ Furthermore, the same group identified the crucial role of redox-active transition metal ions, like copper, in mediating cardiac-aLC toxicity through destabilizing the variable domain. A distinct high level of toxic effects and ROS after cardiac-aLC administration in WT *C. elegans* with copper was observed. This phenomenon was entirely reversed by adding the metal chelator or metal-binding compounds.^120^ Collectively, these findings highlight the specific damage exerted by cardiac-aLCs, even in the absence of Congo red–positive amyloid deposits in the *C. elegans* models (*Table 3*). The data suggest that cytotoxicity is likely mediated by the binding of redox-active metal ions, such as copper. These models present a straightforward, cost-effective, and rapid approach to further investigate the mechanisms underlying cardiac toxicity of LCs in AL amyloidosis.

**Table 3:** Summary of AL *C. elegans* and Zebrafish models

Model strategy	Amyloid deposit formation	Key phenotypes	Advantages	Disadvantages	References
Human aLC transgenic *C. elegans* model	No	Reduced contraction frequency Increased ROS level Mitochondrial ultrastructural damages	Rapid and cost-effective	Phylogenetical distance from vertebrate No amyloid formation	^117^
Primary aLC injection *C. elegans* model	No	Decreased pharyngeal pumping Structure damage on the pharynx	Rapid and cost-effective	Phylogenetical distance from vertebrate No amyloid formation	^118–120^
Human aLC transgenic Zebrafish model	No	Increased cardiac cell death Unaffected lifespan	Rapid and cost-effective Liver-restricted expression	Phylogenetical distance from vertebrate No amyloid formation	^121,122^
Primary aLC injection Zebrafish model	No	Impaired contractile properties Increased oxidative stress Unbalanced autophagic flux	Rapid and cost-effective	Phylogenetical distance from vertebrate No amyloid formation	^73,77,123^

#### 4.2.2 Zebrafish models

Zebrafish, with their more human-like heart structure, high genetic similarity, and exceptional regenerative capacity, have become a valuable model for cardiovascular disease research.^124,125^ In 2013, the first zebrafish model investigating aLC cardiotoxicity was established by directly injecting cardiac-aLCs into the circulation of zebrafish embryos (*Figure 1*).^123^ Despite the absence of Congo red–positive amyloid deposition, zebrafish injected with a pathophysiological dose (100 mg/L) of cardiac-aLCs from AL patients exhibited cardiac contractile dysfunction, increased oxidative stress, activation of non-canonical p38 MAPK signalling, unbalanced autophagic flux, and 100% mortality rate within a 2-week treatment when compared with non-aLCs from MM patients.^75,126^ These findings align with prior evidence showing impaired contractility and up-regulated oxidative stress after injecting aLC mRNA into zebrafish fertilized oocytes.^127^ Pharmacological interventions like p38 MAPK inhibition and rapamycin successfully rescued cardiac dysfunction and attenuated mortality. In 2019, a transgenic zebrafish model was developed to continuously express cardiac-aLCs in the liver at pathophysiological levels (125 mg/L).^121,122^ This model displayed cardiac proteotoxicity, but interestingly, lifespan was not affected, potentially due to observed increased cardiac proliferation in the myocardium. However, the study did not specify which cell types were proliferating, leaving the open question of whether this proliferation truly reflects a compensatory mechanism. Notably, even after 2 years, amyloid deposition was not observed. This raises a concern about the model's ability to accurately reflect the disease state, where amyloid deposition is a hallmark. Additionally, the zebrafish's regenerative ability, particularly in the heart, may mitigate the full impact of aLC proteotoxicity, underscoring a potential limitation in mirroring the human disease state (*Table 3*).

#### 4.2.3 Rodent models

Injecting amyloid precursor proteins or fibrils into mice is a straightforward approach to establish the cardiac amyloidosis model (*Figure 1*). An early attempt focused on intraperitoneal injections of aLCs in mice, which resulted in amyloid deposits in the kidneys. However, this method proved impractical due to the large quantities of purified aLCs required (at the gram scale) and its limited reflection of human AL pathology.^128^

In addition to intraperitoneal injection of aLC, the group of S. Wall has established the AL amyloidomas mouse model by subcutaneously injecting human AL amyloid extracts from patients with AL amyloidosis.^129^ Vascularized and localized tumour-like amyloid deposits, termed amyloidomas, were found in immunocompetent mice but were cleared within ∼14 days of phagocytosis involvement.^129,130^ Later, the effects of liver or spleen injection of *in vitro*-generated AL fibrils derived from the recombinant V_L_-aLCs were also studied.^131^ While these localized amyloidomas do not fully recapitulate AL pathogenesis, this model is valuable for validating the efficacy of novel amyloid-targeting immunotherapy and diagnostic mAbs. Murine monoclonal antibodies (mAb 11-1F4 and mAb 2A4) that bind to LC fibrils regardless of their variable domain subgroup, triggered an Fc-mediated cellular inflammatory response that led to a rapid reduction in the amyloidomas.^112,132^ Radiolabelled mAbs 11-1F4 and 2A4 were developed as amyloid-specific imaging agents, which were readily visible within the amyloidomas through fused small-animal SPECT/CT or small-animal PET/CT images,^112,133^ paving the way for clinical trials to assess their diagnostic potential in various systemic amyloidosis.^115,134–137^ To further enhance amyloid clearance through macrophage uptake, a pan amyloid-reactive peptide (p5) was fused genetically to the LC of the mAbs 11-1F4.^138^ Without interfering the immunologic functionality of the IgG in the context of the p5 fusion, macrophage-mediated uptake of amyloid extracts and purified amyloid fibrils was enhanced, highlighting the therapeutic potential of peptide-antibody fusions for all types of systemic amyloidosis. Collectively, the amyloidomas AL mouse model serves as a robust platform for pre-clinical evaluation of novel therapeutics and the identification of radiotracers for *in vivo* amyloid imaging (*Table 4*). This could enable early disease detection and monitoring in patients through whole-body imaging.

**Table 4:** Summary of AL rodent models

Model strategy	Amyloid deposit formation	Key phenotypes	Advantages	Disadvantages	References
Primary aLC or fibrils injection	Kidney	Not determined	Rapid	Intraperitoneal injections Not recapitulate AL pathogenesis	^128^
	Not determined	Cleared by phagocytosis	Rapid Drug screening possible Novel imaging possible	Limited *ex vivo* AL deposits availability Subcutaneous injection Not recapitulate AL pathogenesis	^129,130^
V-aLC fibrils injection	Liver	Well tolerated to injected fibrils	Rapid Drug screening possible Novel imaging possible	Intrahepatic or intrasplenic injection Not recapitulate AL pathogenesis	^131^
Human LC transgenic model	Stomach	Fibril formation inhibition by doxycycline Neurologic and metabolic phenotype	CMV promoter	Various phenotypes	^139^
	No	Not determined	Hepatocyte-restricted expression	Up to 2 years experimental period	^140^
	Heart and spleen	Increased NT-proBNP level Up-regulation in extracellular matrix remodelling and fibrosis genes	Mouse heavy chain-null background Drug screening possible Novel imaging possible	Amyloid seeding required	^141^

Transgenic approaches have been employed to produce λ aLCs endogenously in mice (*Figure 1*). Amyloid deposition was observed with the random insertion of λ LC under the cytomegalovirus (CMV) promoter, it was limited to the stomach of aged mice without cardiac involvement, suggesting a local acid-mediated aggregation mechanism rather than physiological amyloid formation. Despite not fully simulating human AL pathology, this model demonstrated the ability of doxycycline to inhibit amyloid formation *in vivo*.^139^ However, mice with hepatocyte-restricted expression of λ aLC showed normal lifespan without cytotoxic signs and no amyloid deposition over 2 years, highlighting the challenges in replicating the disease progression of AL amyloidosis.^140^ Similar results were observed when human κ aLC was inserted into the rat Ig κ locus using CRISPR/Cas9 technology.^142^ Although human κ LCs expression was observed in most B and plasma cells, they appeared to be mainly associated with the rat heavy chain to form full-length IgG, resulting in no elevation of the free LC level. To overcome the background influence of endogenous heavy chain, based on the approach to insert κ-aLC into the Ig κ locus, a transgenic mouse model producing high amounts of human free LCs without endogenous heavy chain was developed. Upon the intravenous injection of AL amyloid fibrils from V_L_-aLC, these mice exhibited systemic accumulation of AL amyloid fibrils in the heart and spleen, along with early cardiac dysfunction, like increased levels of NT-proBNP. These fibrils displayed typical pathological features of AL amyloid deposits with most of the classical amyloid accessory proteins (ApoE, ApoA-IV, Vitronectin).^141^ This model, recapitulating key features of systemic AL amyloidosis with cardiac involvement, offers a valuable new tool for AL research and therapeutic development (*Table 4*).

## 5. ATTR cardiomyopathy research models

### 5.1 ATTR *in vitro* cell models

#### 5.1.1 Cardiomyocyte cell models

Several studies have demonstrated the dose-dependent cytotoxic effects of amyloidogenic TTR (WT, Val122Ile, Val30Met, Val20Ile, Leu111Met) on human AC16 cardiomyocytes but not to the stable and non-amyloidogenic Thr119Met TTR (*Table 5*).^143–146^ This toxicity is associated with an increased ROS level and apoptotic cell death. By employing bulk RNAseq and ATACseq to profile the transcriptional and chromatin-level changes in AC16 cardiomyocytes when exposed to WT and variant TTR types (Val122Ile and Leu50Pro) at physiological concentration (200 mg/L), Murphy's group was able to map the earliest cellular signatures upon TTR exposure.^80^ At the transcriptional level, AC16 cells displayed increased expression of genes associated with myogenesis hallmark pathways, specifically when exposed to Val122Ile and Leu50Pro TTR, suggesting a potential protective response against the cellular stress induced by these variant TTRs. Alterations in chromatin-level changes were observed when exposed to variant TTR, which can be effectively reversed by pre-incubation with the kinetic stabilizer Tafamidis. In contrast to the cytotoxic results from AC16 cardiomyocytes, WT and Leu55Pro variant TTR did not show cytotoxicity or influence contractile properties when tested on mouse HL-1 cardiomyocytes and mouse embryonic stem cell (ESC)-derived cardiomyocytes.^147^ On the other hand, TTR fibrils induced under mildly acidic conditions have been shown to impact cell viability, disrupt mitochondrial membrane potential, increase ROS levels and autophagic vacuoles production through extracellular interaction with the cell surface. Functionally, TTR fibrils can prolong the action potential, potentially contributing to the development of arrhythmias and conduction abnormalities frequently observed in patients with ATTR-cardiomyopathy.^148–150^ Next to *in vitro* fibrils, plasma from both ATTRwt and ATTRv patients has been applied to investigate the direct effects on neonatal ventricular cardiomyocytes (NVCMs).^152^ This study showed impaired hypertrophic growth of NVCMs when incubated with plasma from ATTRwt and ATTRv cardiomyopathy patients, yet an enhanced hypertrophic response when cultured with plasma from healthy controls and ATTR-polyneuropathy patients. Although cell hypertrophy is commonly used to assess cellular stress, this contrasting *in vitro* response suggests a potential method for risk prediction in ATTR cardiomyopathy. However, further validation with patient-specific human cardiomyocytes is needed to confirm the clinical utility of this approach.

**Table 5:** Summary of ATTR cardiomyocyte cell models

Amyloid materials	Cell models	Key phenotypes	References
Recombinant WT and Variant TTR supplemented in the cell medium	AC ventricular cardiomyocyte lines	Reduced cell viability Increased ROS level Increased apoptotic cell death Up-regulation of myogenesis hallmark pathway target genes Chromatin-level changes	^80,143–146^
	Mouse HL-1 atrial cardiomyocytes Mouse ESC-cardiomyocytes	Reduced viability upon mTTR Unaltered contractile properties upon L55P TTR	^147^
Acid-induced WT or L55P TTR fibril supplemented in the cell medium	Mouse HL-1 atrial cardiomyocytes Mouse left ventricular myocytes Rat cardiomyocytes H9c2 cells	Reduced viability Decreased mitochondrial membrane potential Increased ROS level Prolonged action potential Increased autophagic vacuoles Cell surface–attached fibrils	^148–150^
Acid-induced WT TTR fibril deposited in the matrix	Neonatal rat ventricular myocytes Adult mouse atrial cardiomyocytes	Decreased contraction and relaxation velocities Decreased force production Prolonged calcium kinetics Sarcomere disruption Decreased intercellular mechanical junctions	^151^
Plasma from ATTR cardiomyopathy patients	Neonatal rat cardiomyocytes	Impaired hypertrophic growth	^152^
Acid-induced WT fibril deposited in the matrix Cell supernatant from the L55P hiPSC-hepatocytes	hiPSC-cardiomyocytes L55P hiPSC-cardiomyocytes	Decreased force production Reduced viability No observable difference for cell morphology Up-regulation in stress-response genes	^151,153^

TTR fibril infiltration is a hallmark of ATTR cardiomyopathy, characterized by the build-up of TTR fibril depositions within the myocardium, leading to thickening and stiffening. To better recapitulate this pathophysiological environment *in vitro*, a new approach has been applied using TTR fibril deposition on the cell plate surface, instead of medium supplementation, to investigate structural and functional changes in various types of cardiomyocytes (NRVMs, hiPSC-CMs, and adult mouse atrial myocytes). Sarcomere disorganization, accompanied by increased ubiquitin localization in the sarcomere was observed, resulting in reduced force generation. Furthermore, these cardiomyocytes exhibited prolonged calcium kinetics and decreased gap junction protein expression, indicative of aberrant electromechanical coupling (*Figure 1*).^151^

#### 5.1.2 hiPS cell models

Induced pluripotent stem cells (iPSCs) provide an unprecedented platform to study the effects of genetic abnormalities and disease progression in ATTR cardiomyopathy. Murphy's group pioneered the development of the first human iPSC model for ATTRv, generating iPSCs from patients with the Leu55Pro mutation and differentiating them into hepatocyte-like cells, cardiomyocytes, and neurons. To replicate the interaction between primary affected tissue types, the cell supernatant from hepatocyte-like cells, containing destabilized, amyloidogenic Leu55Pro TTR, was used to expose differentiated cardiomyocytes and neurons from the same iPSC line. Although no visible amyloid fibrils formed, exposure to this amyloidogenic supernatant resulted in increased oxidative stress and cell death, and the TTR-stabilizing agent diflunisal could mitigate the cytotoxic effects.^153^ Given the genetic heterogeneity and diverse mutation types in ATTRv, a library of iPSC lines has been established from ATTRv patients with various variant TTR genes, ethnicities, and phenotypes. This invaluable resource allows for comprehensive investigation of the underlying pathological mechanisms across different cell types and tissues, paving the way for personalized and targeted therapeutic approaches (*Table 5*).^154–156^

#### 5.1.3 Fibroblast and endothelial cell models

The fibroblast cell line NIH3T3 was shown to endocytose and degrade agitated small WT TTR aggregates, indicated by their co-localization with lysosomes using Lysotracker.^157^ However, this endocytosis phenomenon was not observed in cardiac fibroblasts, indicating the importance of using representative cell types for the specific disease model. Studies by Dittloff *et al*. showed that primary rat cardiac fibroblasts did not take up acid-induced WT TTR fibrils when replated on top of them. Instead, these fibrils disrupted the organization of the cytoskeleton and nucleus, decreased focal adhesions, and reduced cell adhesion. The deposited fibrils also promoted fibroblast proliferation, migration, and the up-regulation of inflammatory genes. These findings suggest that fibroblasts play a complex role in ATTR-cardiomyopathy progression, potentially contributing to inflammation despite their inability to clear fibrils (*Table 6*).^158^

**Table 6:** Summary of ATTR other cell models

Amyloid materials	Cell models	Key phenotypes	References
Aggregated WT and V30M TTR	Mouse NIH3T3 fibroblasts	Endocytosed and degraded aggregated TTR	^157^
Acid-induced WT TTR fibril deposited in the matrix	Primary rat cardiac fibroblasts	Disorganized actin cytoskeleton, Decreased focal adhesion number Flattened nuclear shape Increased proliferation and migration velocity Up-regulation of inflammatory genes	^158^
Recombinant WT and V30M TTR	Primary human umbilical vein endothelial cells	Modulated TNF and IFN pathways Down-regulation of pro-angiogenic genes	^159^
Plasma from ATTR cardiomyopathy patients	primary human aortic endothelial cells (HAECs)	Reduced tissue factor expression upon Tafamidis treatment	^160^
Cell-expressed WT and variant TTR	CHO-K1 HEK cells HepG2 cells	mTTR retained in the ER and degraded Tetrameric TTR secreted normally Platform for ER quality control investigation	^161–164^

The accumulation of TTR amyloid fibrils around blood vessels, including capillaries, can lead to a narrowing of the vessel lumen, disruption of capillary structure, and a decrease in capillary density.^165–167^ Val30Met TTR has been shown to not only modulate interferon (IFN) and tumour necrosis factor (TNF) pathways in human umbilical vein endothelial cells, but also trigger apoptosis and hinder the migration of these cells. This suggests that Val30Met TTR may disrupt the normal function of endothelial cells, potentially contributing to vascular complications in affected patients (*Table 6*).^159^ In another study, primary human aortic endothelial cells (HAECs) were exposed to plasma from ATTRwt cardiomyopathy patients, with and without Tafamidis treatment. While no significant cytotoxic effects were observed, HAECs incubated with Tafamidis-treated plasma displayed reduced tissue factor expression, a key trigger in the coagulation cascade, suggesting a novel protective mechanism of Tafamidis independent of TTR stabilization.^160^

#### 5.1.4 TTR overexpression cell models

The ER stress and the UPR signalling pathways are crucial in regulating ER quality control by preventing the trafficking of non-native proteins and degrading misfolded proteins in the ER through mechanisms including ERAD and ER-phagy.^168^ However, amyloidogenic WT and variant TTR, and aLCs are known to be efficiently secreted by mammalian cells, despite their tendency to misfold.^169^ However, the mechanisms allowing this evasion of ER quality control are still largely unknown.

By overexpressing WT, non-amyloidogenic and amyloidogenic TTR variants in CHO-K1 cells, both in their tetrameric and monomeric forms, Sato *et al*. observed that amyloidogenic TTR monomers were retained in the ER and subsequently degraded by ERAD (*Figure 1*). In contrast, tetrameric WT TTR and amyloidogenic TTR variants were secreted normally in the cell media. This study suggests that the ER quality control system differentially regulates WT and variant TTR tetramers and their monomeric counterparts.^161^ While misfolded amyloidogenic monomeric TTRs are efficiently recognized and targeted for degradation, WT and amyloidogenic TTR variants are likely to evade this process, possibly due to their tetrameric structure within the ER. This allows them to bypass ER quality control and be secreted, ultimately contributing to amyloid fibril formation in the extracellular space. On the other hand, ATF6, the crucial UPR-associated transcription factor, has been shown to preferentially reduce secretion and subsequent aggregation of variant TTRs.^162,163^ ATF6 activation in WT and Ala25Thr TTR overexpressed HEK and HepG2 cells showed TTR retention within the ER through increased interactions with ATF6-regulated ER chaperones including the ER HSP70 BiP and protein disulfide isomerase PDIA4 (*Table 6*).^164^ A similar observation was done by employing Leu55Pro ATTRv patient-specific iPSC-derived hepatocyte-like cells. Single-cell RNAseq data showed that increased expression of multiple UPR-regulated ER proteostasis factors and activation of the ATF6 signalling preferentially reduced the variant TTR expression, indicating that secreted Leu55Pro TTR in hepatic cells disrupted the proteostasis environment and in turn triggered UPR activation.^170^ Additionally, similar results have been found in aLC-overexpressed HEK cells, in which activation of ATF6 selectively attenuated secretion of aLC (*Table 2*).^99^ These findings highlight a potential therapeutic approach for AL and ATTR by targeting the modulation of the ER quality control pathways, such as pharmacologic ER proteostasis regulators, to alleviate destabilized, aLC and TTR secretion and induced proteotoxicity.^171^

### 5.2 ATTR *in vivo* animal models

#### 5.2.1 *Caenorhabditis elegans* models

To date, *C. elegans* has not been used to directly model ATTR cardiomyopathy. In 2018, transgenic *C. elegans* models expressing human WT, Val30Met, or Asp18Gly TTR in the body-wall muscle were developed to investigate the non-autonomous neuronal cells death. These models exhibited TTR aggregation and impaired pain sensation, accompanied by altered neuronal morphology. Unfortunately, this study did not assess whether the observed TTR aggregates were Congo red positive to confirm amyloid fibril formation, nor did it evaluate pharynx performance as an indicator of cardiac function.^172^ While this model provides insights into the neurotoxic effects of TTR aggregation, its relevance to ATTR cardiomyopathy remains to be explored.

#### 5.2.2 Rodent models

Several transgenic mouse models have been developed to investigate ATTR amyloidosis (*Figure 1*, *Table 7*). The first humanized WT transgenic mouse model retaining the endogenous mouse WT TTR^173,174^ showed signs of cardiac inflammation and immune response at a young age of 3 months before any cardiac amyloid deposition was observed, and half of the transgenic male mice eventually developed cardiac amyloid deposits resembling ATTRwt-CM in older mice (24 months). A closer look at gene expression revealed that hearts with confirmed amyloid deposits had a distinct profile, characterized by up-regulated stress response genes, increased mitochondrial gene transcription, and elevated expression of genes linked to apoptosis. Intriguingly, transgenic mice without cardiac amyloid deposition exhibited increased chaperone gene expression and an activated UPR in the liver, which were absent in mice with cardiac amyloid deposition, suggesting a potential link between the compromised hepatic proteostatic capacity and the formation of cardiac amyloid deposition. The diminished ability of the liver to maintain protein balance might lead to more misfolded human WT TTR escaping into the circulation, ultimately reaching the heart and contributing to amyloid formation there.

**Table 7:** Summary of ATTR rodent models

Model strategy	Amyloid deposit formation	Key phenotypes	Advantages	Disadvantages	References
Human WT transgenic model	Heart and kidney	Cardiac inflammation and immune response in 3-month mice Up-regulated stress response, mitochondrial, apoptosis genes	Naturally cardiac amyloid deposits development Drug screening possible Novel imaging possible	Up to 2 years experimental period Only 20% developed into amyloid deposits Mouse WT TTR retained	^173,174^
Human L55P transgenic model	Gastrointestinal tract and skin	Increased cytotoxic stress	Mouse TTR-null background	No amyloid deposition in the heart and nerve system	^175^
Human V30M transgenic model	No	TTR deposition within the heart Macrophage activation	Mouse TTR-null background TTR deposition in the gastrointestinal tract, skin, and peripheral and autonomic nervous system	Up to 2 years experimental period No amyloid deposition development	^157,176,177^
Human S52P transgenic model	Heart and tongue	Amyloid deposits containing full-length TTR and the residue 49–127 cleavage fragment	Naturally cardiac amyloid deposits development Drug screening possible Novel imaging possible	Amyloid seeding required	^178^
*Ex vivo* TTR deposits injection	Not determined	TTR deposits removed	Rapid Drug screening possible Novel imaging possible	*Ex vivo* TTR deposits availability Subcutaneous injection Not recapitulate ATTR pathogenesis	^179^
Acid-induced WT TTR fibrils injection	Not determined	TTR fibrils removed Improved cardiac performance	Rapid Drug screening possible Novel imaging possible PyP scintigraphy available ECG recording	Intracardiac injection Not recapitulate ATTR pathogenesis	^150^

A humanized Leu55Pro transgenic mouse model lacking endogenous mouse TTR showed amyloid fibril deposition in the gastrointestinal tract and skin starting at 4–8 months of age, but notably not in the heart.^175^ In contrast, a similar Leu55Pro transgenic model with intact endogenous mouse TTR exhibited no amyloid deposition till 24 months, highlighting the importance of knocking out murine WT TTR to accelerate amyloidogenesis.^173,180^ Another model, expressing human Val30Met TTR in a mouse TTR null and heat shock transcription factor 1 (hsf1) background, has been extensively utilized for the characterization and modulation of neurological features of Val30Met ATTRv amyloidosis.^176,181–184^ Despite the presence of human Val30Met TTR within the heart, mainly in the intercellular space, no amyloid deposits were observed in cardiac tissues even after 20 months of culture, suggesting a potential resistance to cardiac amyloid deposition in this model. Additionally, human TTR binding to mouse RBP4 resulted in different kinetic and thermodynamic stability profiles of TTR tetramers. By replacing the mouse RBP4 with a humanized counterpart, amyloid deposition in the heart was observed at 24 months of age, suggesting that the TTR-RBP4 interaction is important for amyloid deposition.^185^ Furthermore, TTR endocytosis by fibroblasts, previously observed in mouse skin fibroblasts and patient skin autopsies,^157^ was not observed in the transgenic mouse heart. Co-localization of macrophages with human Val30Met TTR, along with increased galectin-3 expression, indicated macrophage activation in response to the presence of human Val30Met TTR in the cardiac tissue.^177^ This model, while valuable for studying neurological aspects, may not fully recapitulate the cardiac manifestations of Val30Met ATTRv amyloidosis. Lastly, Slamova *et al*.^178^ developed a mouse strain with transgenic expression of human Ser52Pro TTR that can form significant amyloid deposits in the heart and tongue after 4–11 months, achieved by seeding with amyloid extracted from a Ser52Pro ATTR amyloidosis patient. This model is distinctive due to the presence of TTR amyloid deposits containing both full-length TTR and the residue 49–127 cleavage fragments, highlighting the involvement of proteolysis in TTR fibril formation. The enrichment of the serine protease plasmin in amyloid deposits further supports the mechano-enzymatic hypothesis proposing that TTR fragment-induced fibrillogenesis is enhanced after proteolysis.^65,67,68^ These results suggest that abnormal proteolytic activity may be instrumental in facilitating TTR fibril formation. This ATTRv-CM model holds immense potential for investigating the initiation mechanisms of ATTR deposition and serves as a valuable tool for developing novel treatments.

In 2018, a pioneering study demonstrated the long-term efficacy of an LNP-based CRISPR/Cas9 delivery system (NTLA-2001) in knocking down serum TTR levels in normal mice. This proof-of-concept study showed remarkable long-term effectiveness (more than 12 months), achieving a >97% reduction in serum mouse TTR levels with a single administration.^186^ Subsequent clinical trials in patients with ATTRv polyneuropathy showed a dose-dependent, durable TTR reduction with minimal side effects, solidifying *in vivo* CRISPR/Cas9-mediated genome editing as a promising therapeutic avenue for ATTRv.^187^ Also, studies using mAbs that specifically targeted disease-associated WT and variant ATTR aggregates in normal mice have yielded encouraging results. For instance, the mAb NI301A effectively removed patient-derived ATTR fibrils that were grafted in mice through macrophage clearance.^179^ In another study, a short-term rat model was created by injecting *in vitro* aggregated TTR directly into the apex of the heart, showcasing the potential of a different IgG1 mAb. This mAb not only improved cardiac function but also promoted the degradation of aggregated TTR, as evidenced by reduced PYP scintigraphy signals.^150^ These studies collectively highlight the potential of both gene editing and immunotherapy in combating ATTR amyloidosis (*Table 7*). The rapid and efficient mouse models with direct amyloid injection provide a valuable platform for evaluating future therapeutic candidates, further accelerating the development of effective treatments for ATTR amyloidosis.

#### 5.2.3 Non-human primate models

Spontaneous cases of both ATTRwt and Val122Ile ATTRv have been reported in vervet monkeys, exhibiting similar clinical phenotypes observed in human ATTR-cardiomyopathy patients, such as arrhythmia and decreased ejection fraction in aged vervet monkeys.^188–190^ These findings highlight the potential of vervet monkeys as a valuable animal model for studying ATTR amyloidosis, given the similarities in disease presentation and genetic predisposition.

## 6. Conclusions and perspectives

Cardiac amyloidosis, primarily encompassing AL and ATTR types, is fundamentally driven by the misfolding and aggregation of precursor proteins, immunoglobulin LC and TTR, into amyloid fibrils. While genetic mutations and amino acid sequence alterations play pivotal roles in initiating fibril formation, particularly during the lag phase of fibrillogenesis,^191^ proteolysis emerges as another significant contributor. The heterogeneous LC sequences in AL amyloidosis and variant TTR in ATTRv amyloidosis are inherently prone to misfolding and aggregation.^16–26^ Proteolytic cleavage,^19,33–35,61,62^ which occurs to generate fragments of LC and TTR, also contributes to accelerating amyloid nucleus formation, highlighting the importance of both intrinsic protein instability and enzymatic processing in amyloid fibril formation. Due to the difficulties in recapitulating the lag phase of amyloid fibril formation in research models, the ability to bypass the lengthy lag phase using amyloid seeds, either patient-derived or *in vitro* generated,^56,58,141,178^ has significantly advanced research by enabling the rapid recapitulation of fibril formation in experimental settings, effectively addressing a major challenge in studying the chronic disease progression.

Cell models have provided critical insights into the cytotoxic mechanisms of both AL and ATTR amyloidosis. In AL amyloidosis, a consensus has gradually been reached that both aLC and LC fibrils can reduce cell viability by increasing oxidative stress,^72,76^ but their underlying proteotoxic mechanisms appear to be different. Soluble aLCs primarily disrupt lysosomal autophagy^73^ and induce ER stress,^192^ while LC fibrils trigger cell growth arrest, metabolic impairment, and innate immune responses.^76,81,83^ Similarly, in ATTR amyloidosis, though the exact toxic species remains debated, evidence suggests that both soluble TTR oligomers^153,193^ and TTR fibrils^147–151,158^ contribute to cellular dysfunction. TTR fibrils, in particular, induce effects similar to LC fibrils, including reduced cell viability, increased oxidative stress, and prolonged calcium handling.^148,151^ However, the use of acid-induced TTR fibrils presents a methodological challenge due to their structural differences from physiological fibrils.^45,68^ Although *in vitro*-generated fibrils exhibit native-like β-sheet structures, they often fail to replicate the complex molecular organization and surface properties of patient-derived fibrils,^28,194,195^ exhibiting less stability to proteolysis.^196^ This divergence is critical because cellular interactions depend heavily on exposed fibril regions. Therefore, acid-induced fibrils, formed under non-physiological conditions, may exhibit altered molecular organization and surface properties, potentially leading to inaccurate interpretations of their biological interactions.

Animal models, including *C. elegans*, zebrafish, and rodents, have been instrumental in studying cardiac amyloidosis, though each presents unique challenges. Acute injection of aLCs or fibrils, while straightforward, fails to replicate the chronic progression of the disease.^118,119,123,128,129^ Transgenic models, which continuously produce amyloid precursor proteins, offer a more physiologically relevant approach, but often require amyloid seeding to induce cardiac deposition.^141,178^ The limited observation of fragmented precursor proteins also suggests minimal effect of proteolytic rearrangement compared with what is observed in patients. AL animal models have consistently demonstrated the direct toxic effects of soluble aLCs on cardiac cells, including apoptosis, oxidative stress, mitochondrial dysfunction, impaired contractility and calcium handling, lysosomal dysfunction, and disrupted autophagy, reinforcing their role in cardiac damage. ATTR animal models, particularly transgenic mice, have provided insights into the role of proteolysis and hepatic proteostasis in disease pathogenesis.^174,178^ However, the difficulty in achieving robust cardiac amyloid deposition without seeding underscores the complexity of replicating the complete disease process *in vivo*, highlighting the need for continued refinement of these models to better reflect human pathology.

### 6.1 Future perspectives

**Figure 2:**
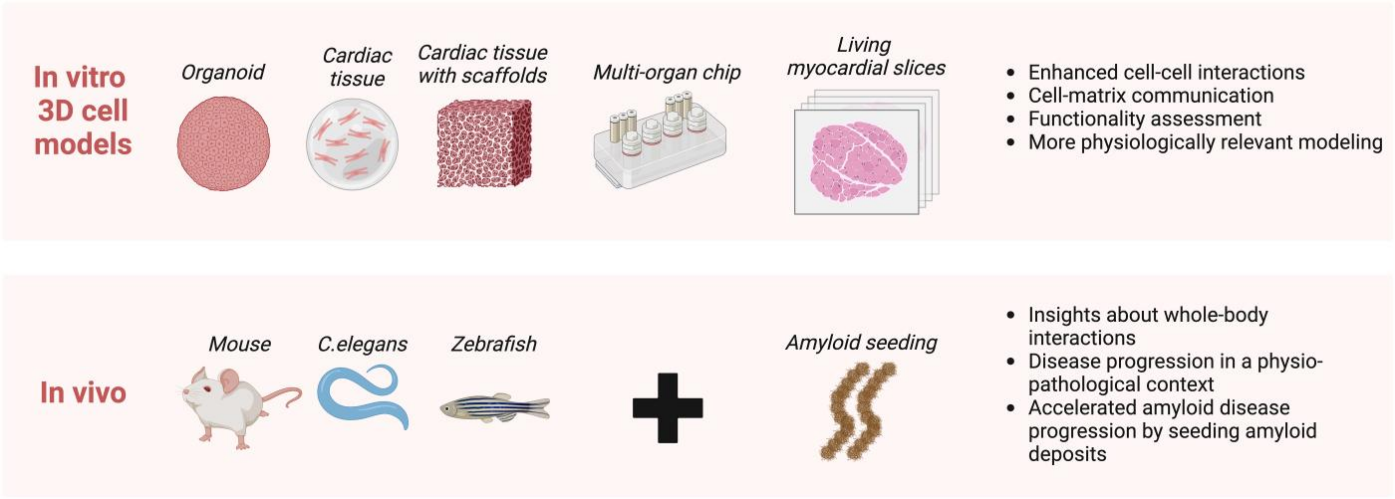
Future perspectives in cardiac amyloidosis disease modelling. *In vitro* 3D cell models offer improved physiological relevance compared with traditional 2D cell culture. These models include organoids, cardiac tissue, engineered cardiac tissue with scaffolds, multiorgan-on-a-chip technology, and explanted living myocardial slices. *In vivo* animal models continue to be essential for investigating whole-body disease progression. The use of amyloid seeds in these models is increasingly important for accelerating disease onset. Created with Biorender.com.

Traditional 2D static cell culture lacks the complex cell-to-cell and cell-to-ECM interactions that occur in the native heart tissue. Therefore, developing more sophisticated 3D models, such as cardiac organoids,^197^ engineered cardiac tissue,^198,199^ and living myocardial slices,^200–202^ is essential for advancing disease understanding and drug discovery. Also, heart-on-a-chip technology,^203^ integrating cardiac tissue with microfluidics, offers further potential by replicating key elements of the human microphysiological system. Given the pathology of cardiac amyloidosis, where LCs and TTR are secreted from distant organs like the bone marrow and liver, incorporating blood flow simulation to deliver amyloidogenic proteins to cardiac tissues would significantly enhance the physiological relevance of these models (*Figure 2*).

Amyloid seeding with *ex vivo* amyloid extracts has advanced transgenic models (*Figure 2*).^141,178^ but the complex composition of these extracts, containing fibrils and accessory proteins like SAP and ECM components, necessitates further investigation. These amyloid accessory proteins, which frequently accompany amyloid fibrils within amyloid deposits, play a role in accelerating amyloid aggregation, cell–amyloid interactions, and amyloid degradation.^204–208^ For example, SAP removal has shown promise in clearing amyloid deposits in both murine and human studies.^206,209^ In addition, the lifespan of these transgenic models has not been fully reported. Although an increase of NT-proBNP was observed in an AL mouse model,^141^ their ability to model the disease's characteristic reduction in survival is undefined. This lack of lifespan data is a critical limitation, as it prevents accurate assessment of how well these models reflect the progressive feature of cardiac amyloidosis. Therefore, addressing these limitations is crucial to refine animal and cell models and improve early diagnosis of cardiac amyloidosis.

In conclusion, continuous efforts are needed to develop more advanced and comprehensive models that accurately reflect the intricate interplay of factors involved in cardiac amyloidosis. Such models will undoubtedly play a crucial role in advancing our understanding and developing innovative diagnostic and therapeutic strategies.

## Data Availability

No new data were generated or analysed in support of this research.

## References

[1] Wechalekar AD, Gillmore JD, Hawkins PN. Systemic amyloidosis. Lancet 2016;387:2641–2654. 26719234 10.1016/S0140-6736(15)01274-X

[2] Riek R, Eisenberg DS. The activities of amyloids from a structural perspective. Nature 2016;539:227–235. 27830791 10.1038/nature20416

[3] Buxbaum JN, Eisenberg DS, Fändrich M, McPhail ED, Merlini G, Saraiva MJM, Sekijima Y, Westermark P. Amyloid nomenclature 2024: update, novel proteins, and recommendations by the International Society of Amyloidosis (ISA) Nomenclature Committee. Amyloid 2024;31:249–256. 39350582 10.1080/13506129.2024.2405948

[4] Buxbaum JN, Ruberg FL. Transthyretin V122I (pV142I)* cardiac amyloidosis: an age-dependent autosomal dominant cardiomyopathy too common to be overlooked as a cause of significant heart disease in elderly African Americans. Genet Med 2017;19:733–742. 28102864 10.1038/gim.2016.200 PMC5509498

[5] Oerlemans MIFJ, Rutten KHG, Minnema MC, Raymakers RAP, Asselbergs FW, de Jonge N. Cardiac amyloidosis: the need for early diagnosis. Neth Heart J 2019;27:525–536. 31359320 10.1007/s12471-019-1299-1 PMC6823341

[6] Brons M, Muller SA, Rutten FH, Meer Mvd, Vrancken AFJE, Minnema MC, Baas AF, Asselbergs FW, Oerlemans MIFJ. Evaluation of the cardiac amyloidosis clinical pathway implementation: a real-world experience. Eur Heart J Open 2022;2:oeac011. 35919127 10.1093/ehjopen/oeac011 PMC9242028

[7] McCausland KL, White MK, Guthrie SD, Quock T, Finkel M, Lousada I, Bayliss MS. Light chain (AL) amyloidosis: the journey to diagnosis. Patient 2018;11:207–216. 28808991 10.1007/s40271-017-0273-5 PMC5845075

[8] Oubari S, Naser E, Papathanasiou M, Luedike P, Hagenacker T, Thimm A, Rischpler C, Kessler L, Kimmich C, Hegenbart U, Schönland S, Rassaf T, Reinhardt HC, Jöckel K-H, Dürig J, Dührsen U, Carpinteiro A. Impact of time to diagnosis on Mayo stages, treatment outcome, and survival in patients with AL amyloidosis and cardiac involvement. Eur J Haematol 2021;107:449–457. 34185342 10.1111/ejh.13681

[9] Merlini G, Dispenzieri A, Sanchorawala V, Schönland SO, Palladini G, Hawkins PN, Gertz MA. Systemic immunoglobulin light chain amyloidosis. Nat Rev Dis Primers 2018;4:38. 30361521 10.1038/s41572-018-0034-3

[10] Merlini G, Bellotti V. Molecular mechanisms of amyloidosis. N Engl J Med 2003;349:583–596. 12904524 10.1056/NEJMra023144

[11] Falk RH, Alexander KM, Liao R, Dorbala S. AL (light-chain) cardiac amyloidosis: a review of diagnosis and therapy. J Am Coll Cardiol 2016;68:1323–1341. 27634125 10.1016/j.jacc.2016.06.053

[12] Blancas-Mejia LM, Misra P, Dick CJ, Cooper SA, Redhage KR, Bergman MR, Jordan TL, Maar K, Ramirez-Alvarado M. Immunoglobulin light chain amyloid aggregation. Chem Commun 2018;54:10664–10674. 10.1039/c8cc04396e PMC6148388 30087961

[13] Morgan GJ, Wall JS. The process of amyloid formation due to monoclonal immunoglobulins. Hematol Oncol Clin North Am 2020;34:1041–1054. 33099422 10.1016/j.hoc.2020.07.003

[14] Absmeier RM, Rottenaicher GJ, Svilenov HL, Kazman P, Buchner J. Antibodies gone bad—the molecular mechanism of light chain amyloidosis. FEBS J 2023;290:1398–1419. 35122394 10.1111/febs.16390

[15] Mishra AK, Mariuzza RA. Insights into the structural basis of antibody affinity maturation from next-generation sequencing. Front Immunol 2018;9:117. 29449843 10.3389/fimmu.2018.00117 PMC5799246

[16] Kim Y, Wall JS, Meyer J, Murphy C, Randolph TW, Manning MC, Solomon A, Carpenter JF. Thermodynamic modulation of light chain amyloid fibril formation. J Biol Chem 2000;275:1570–1574. 10636846 10.1074/jbc.275.3.1570

[17] Wall J, Schell M, Murphy C, Hrncic R, Stevens FJ, Solomon A. Thermodynamic instability of human lambda 6 light chains: correlation with fibrillogenicity. Biochemistry 1999;38:14101–14108. 10529258 10.1021/bi991131j

[18] Raffen R, Dieckman LJ, Szpunar M, Wunschl C, Pokkuluri PR, Dave P, Wilkins Stevens P, Cai X, Schiffer M, Stevens FJ. Physicochemical consequences of amino acid variations that contribute to fibril formation by immunoglobulin light chains. Protein Sci 1999;8:509–517. 10091653 10.1110/ps.8.3.509 PMC2144278

[19] Kazman P, Vielberg M-T, Pulido Cendales MD, Hunziger L, Weber B, Hegenbart U, Zacharias M, Köhler R, Schönland S, Groll M, Buchner J. Fatal amyloid formation in a patient’s antibody light chain is caused by a single point mutation. eLife 2020;9:e52300. 32151314 10.7554/eLife.52300 PMC7064341

[20] Blancas-Mejía LM, Tischer A, Thompson JR, Tai J, Wang L, Auton M, Ramirez-Alvarado M. Kinetic control in protein folding for light chain amyloidosis and the differential effects of somatic mutations. J Mol Biol 2014;426:347–361. 24157440 10.1016/j.jmb.2013.10.016 PMC3892967

[21] Baden EM, Randles EG, Aboagye AK, Thompson JR, Ramirez-Alvarado M. Structural insights into the role of mutations in amyloidogenesis. J Biol Chem 2008;283:30950–30956. 18768467 10.1074/jbc.M804822200 PMC2576559

[22] Randles EG, Thompson JR, Martin DJ, Ramirez-Alvarado M. Structural alterations within native amyloidogenic immunoglobulin light chains. J Mol Biol 2009;389:199–210. 19361523 10.1016/j.jmb.2009.04.010 PMC2840394

[23] Rennella E, Morgan GJ, Kelly JW, Kay LE. Role of domain interactions in the aggregation of full-length immunoglobulin light chains. Proc Natl Acad Sci U S A 2019;116:854–863. 30598439 10.1073/pnas.1817538116 PMC6338840

[24] Brumshtein B, Esswein SR, Landau M, Ryan CM, Whitelegge JP, Phillips ML, Cascio D, Sawaya MR, Eisenberg DS. Formation of amyloid fibers by monomeric light chain variable domains. J Biol Chem 2014;289:27513–27525. 25138218 10.1074/jbc.M114.585638 PMC4183792

[25] Baden EM, Owen BAL, Peterson FC, Volkman BF, Ramirez-Alvarado M, Thompson JR. Altered dimer interface decreases stability in an amyloidogenic protein. J Biol Chem 2008;283:15853–15860. 18400753 10.1074/jbc.M705347200 PMC2414275

[26] Morgan GJ, Kelly JW. The kinetic stability of a full-length antibody light chain dimer determines whether endoproteolysis can release amyloidogenic variable domains. J Mol Biol 2016;428:4280–4297. 27569045 10.1016/j.jmb.2016.08.021 PMC5065776

[27] Swuec P, Lavatelli F, Tasaki M, Paissoni C, Rognoni P, Maritan M, Brambilla F, Milani P, Mauri P, Camilloni C, Palladini G, Merlini G, Ricagno S, Bolognesi M. Cryo-EM structure of cardiac amyloid fibrils from an immunoglobulin light chain AL amyloidosis patient. Nat Commun 2019;10:1269. 30894521 10.1038/s41467-019-09133-w PMC6427027

[28] Radamaker L, Lin Y-H, Annamalai K, Huhn S, Hegenbart U, Schönland SO, Fritz G, Schmidt M, Fändrich M. Cryo-EM structure of a light chain-derived amyloid fibril from a patient with systemic AL amyloidosis. Nat Commun 2019;10:1103. 30894526 10.1038/s41467-019-09032-0 PMC6427026

[29] Xu L, Su Y. Genetic pathogenesis of immunoglobulin light chain amyloidosis: basic characteristics and clinical applications. Exp Hematol Oncol 2021;10:43. 34284823 10.1186/s40164-021-00236-z PMC8290569

[30] Nishita M, Park S-Y, Nishio T, Kamizaki K, Wang Z, Tamada K, Takumi T, Hashimoto R, Otani H, Pazour GJ, Hsu VW, Minami Y. Ror2 signaling regulates Golgi structure and transport through IFT20 for tumor invasiveness. Sci Rep 2017;7:1. 28127051 10.1038/s41598-016-0028-x PMC5428335

[31] Bourne PC, Ramsland PA, Shan L, Fan ZC, DeWitt CR, Shultz BB, Terzyan SS, Moomaw CR, Slaughter CA, Guddat LW, Edmundson AB. Three-dimensional structure of an immunoglobulin light-chain dimer with amyloidogenic properties. Acta Crystallogr D Biol Crystallogr 2002;58:815–823. 11976493 10.1107/s0907444902004183

[32] Weiss BM, Hebreo J, Cordaro DV, Roschewski MJ, Baker TP, Abbott KC, Olson SW. Increased serum free light chains precede the presentation of immunoglobulin light chain amyloidosis. J Clin Oncol 2014;32:2699–2704. 25024082 10.1200/JCO.2013.50.0892 PMC4145182

[33] Annamalai K, Gührs K-H, Koehler R, Schmidt M, Michel H, Loos C, Gaffney PM, Sigurdson CJ, Hegenbart U, Schönland S, Fändrich M. Polymorphism of amyloid fibrils *in vivo*. Angew Chem Int Ed 2016;55:4822–4825. 10.1002/anie.201511524 PMC4864496 26954430

[34] Lavatelli F, Mazzini G, Ricagno S, Iavarone F, Rognoni P, Milani P, Nuvolone M, Swuec P, Caminito S, Tasaki M, Chaves-Sanjuan A, Urbani A, Merlini G, Palladini G. Mass spectrometry characterization of light chain fragmentation sites in cardiac AL amyloidosis: insights into the timing of proteolysis. J Biol Chem 2020;295:16572–16584. 32952127 10.1074/jbc.RA120.013461 PMC7864057

[35] Lavatelli F, Perlman DH, Spencer B, Prokaeva T, McComb ME, Théberge R, Connors LH, Bellotti V, Seldin DC, Merlini G, Skinner M, Costello CE. Amyloidogenic and associated proteins in systemic amyloidosis proteome of adipose tissue. Mol Cell Proteomics 2008;7:1570–1583. 18474516 10.1074/mcp.M700545-MCP200 PMC2494907

[36] Weber B, Hora M, Kazman P, Pradhan T, Rührnößl F, Reif B, Buchner J. Domain interactions determine the amyloidogenicity of antibody light chain mutants. J Mol Biol 2020;432:6187–6199. 33058870 10.1016/j.jmb.2020.10.005

[37] Kazman P, Absmeier RM, Engelhardt H, Buchner J. Dissection of the amyloid formation pathway in AL amyloidosis. Nat Commun 2021;12:6516. 34764275 10.1038/s41467-021-26845-0 PMC8585945

[38] Klimtchuk ES, Gursky O, Patel RS, Laporte KL, Connors LH, Skinner M, Seldin DC. The critical role of the constant region in thermal stability and aggregation of amyloidogenic immunoglobulin light chain. Biochemistry 2010;49:9848–9857. 20936823 10.1021/bi101351c PMC4080313

[39] Solomon A, Weiss DT, Murphy CL, Hrncic R, Wall JS, Schell M. Light chain-associated amyloid deposits comprised of a novel kappa constant domain. Proc Natl Acad Sci U S A 1998;95:9547–9551. 9689117 10.1073/pnas.95.16.9547 PMC21375

[40] Klafki HW, Kratzin HD, Pick AI, Eckart K, Karas M, Hilschmann N. Complete amino acid sequence determinations demonstrate identity of the urinary Bence Jones protein (BJP-DIA) and the amyloid fibril protein (AL-DIA) in a case of AL-amyloidosis. Biochemistry 1992;31:3265–3272. 1554711 10.1021/bi00127a031

[41] Hanson JLS, Arvanitis M, Koch CM, Berk JL, Ruberg FL, Prokaeva T, Connors LH. Use of serum transthyretin as a prognostic indicator and predictor of outcome in cardiac amyloid disease associated with wild-type transthyretin. Circ Heart Fail 2018;11:e004000. 29449366 10.1161/CIRCHEARTFAILURE.117.004000 PMC5819619

[42] Lindquist SL, Kelly JW. Chemical and biological approaches for adapting proteostasis to ameliorate protein misfolding and aggregation diseases: progress and prognosis. Cold Spring Harb Perspect Biol 2011;3:a004507. 21900404 10.1101/cshperspect.a004507 PMC3225948

[43] Naidoo N . ER and aging-protein folding and the ER stress response. Ageing Res Rev 2009;8:150–159. 19491040 10.1016/j.arr.2009.03.001

[44] Jiang X, Buxbaum JN, Kelly JW. The V122I cardiomyopathy variant of transthyretin increases the velocity of rate-limiting tetramer dissociation, resulting in accelerated amyloidosis. Proc Natl Acad Sci U S A 2001;98:14943–14948. 11752443 10.1073/pnas.261419998 PMC64963

[45] Buxbaum JN, Dispenzieri A, Eisenberg DS, Fändrich M, Merlini G, Saraiva MJM, Sekijima Y, Westermark P. Amyloid nomenclature 2022: update, novel proteins, and recommendations by the International Society of Amyloidosis (ISA) Nomenclature Committee. Amyloid 2022;29:213–219. 36420821 10.1080/13506129.2022.2147636

[46] Lobato L . Portuguese-type amyloidosis (transthyretin amyloidosis, ATTR V30M). J Nephrol 2003;16:438–442. 12832749

[47] Muller SA, Peiró-Aventin B, Biagioni G, Tini G, Saturi G, Kronberger C, Achten A, Dobner S, Te Rijdt WP, Gasperetti A, Te Riele ASJM, Varrà GG, Ponziani A, Hirsch A, Porcari A, van der Meer M, Zampieri M, van der Harst P, Kammerlander A, Biagini E, van Tintelen J, Barbato E, Asselbergs FW, Menale S, Gräni C, Merlo M, Michels M, Knackstedt C, Nitsche C, Longhi S, Musumeci B, Cappelli F, Garcia-Pavia P, Oerlemans MIFJ. Evaluation of the 2021 ESC recommendations for family screening in hereditary transthyretin cardiac amyloidosis. Eur J Heart Fail 2024;26(9):2025–2034. 38887861 10.1002/ejhf.3339

[48] Zeldenrust SR . Genotype--phenotype correlation in FAP. Amyloid 2012;19(Suppl 1):22–24. 22620962 10.3109/13506129.2012.665400

[49] Ruberg FL, Maurer MS, Judge DP, Zeldenrust S, Skinner M, Kim AY, Falk RH, Cheung KN, Patel AR, Pano A, Packman J, Grogan DR. Prospective evaluation of the morbidity and mortality of wild-type and V122I mutant transthyretin amyloid cardiomyopathy: the Transthyretin Amyloidosis Cardiac Study (TRACS). Am Heart J 2012;164:222–228.e1. 22877808 10.1016/j.ahj.2012.04.015

[50] Foss TR, Wiseman RL, Kelly JW. The pathway by which the tetrameric protein transthyretin dissociates. Biochemistry 2005;44:15525–15533. 16300401 10.1021/bi051608t

[51] Hamilton JA, Benson MD. Transthyretin: a review from a structural perspective. Cell Mol Life Sci 2001;58:1491–1521. 11693529 10.1007/PL00000791 PMC11337270

[52] Kelly JW . The alternative conformations of amyloidogenic proteins and their multi-step assembly pathways. Curr Opin Struct Biol 1998;8:101–106. 9519302 10.1016/s0959-440x(98)80016-x

[53] Hurshman AR, White JT, Powers ET, Kelly JW. Transthyretin aggregation under partially denaturing conditions is a downhill polymerization. Biochemistry 2004;43:7365–7381. 15182180 10.1021/bi049621l

[54] Saelices L, Johnson LM, Liang WY, Sawaya MR, Cascio D, Ruchala P, Whitelegge J, Jiang L, Riek R, Eisenberg DS. Uncovering the mechanism of aggregation of human transthyretin. J Biol Chem 2015;290:28932–28943. 26459562 10.1074/jbc.M115.659912 PMC4661406

[55] Jiang X, Smith CS, Petrassi HM, Hammarström P, White JT, Sacchettini JC, Kelly JW. An engineered transthyretin monomer that is nonamyloidogenic, unless it is partially denatured? Biochemistry 2001;40:11442–11452. 11560492 10.1021/bi011194d

[56] Saelices L, Chung K, Lee JH, Cohn W, Whitelegge JP, Benson MD, Eisenberg DS. Amyloid seeding of transthyretin by *ex vivo* cardiac fibrils and its inhibition. Proc Natl Acad Sci U S A 2018;115:E6741–E6750. 29954863 10.1073/pnas.1805131115 PMC6055172

[57] Morfino P, Aimo A, Panichella G, Rapezzi C, Emdin M. Amyloid seeding as a disease mechanism and treatment target in transthyretin cardiac amyloidosis. Heart Fail Rev 2022;27:2187–2200. 35386059 10.1007/s10741-022-10237-7 PMC9546974

[58] Nguyen BA, Singh V, Afrin S, Yakubovska A, Wang L, Ahmed Y, Pedretti R, Fernandez-Ramirez MDC, Singh P, Pękała M, Cabrera Hernandez LO, Kumar S, Lemoff A, Gonzalez-Prieto R, Sawaya MR, Eisenberg DS, Benson MD, Saelices L. Structural polymorphism of amyloid fibrils in ATTR amyloidosis revealed by cryo-electron microscopy. Nat Commun 2024;15:581. 38233397 10.1038/s41467-024-44820-3 PMC10794703

[59] Ripoll-Vera T, Alvarez J, Buades J, Cisneros E, Gomez Y, Melia C, Ferrer A, Losada I, Gonzalez J, Uson M, Figuerola A. Cardiac involvement after liver transplantation in patients with Val30Met transthyretin amyloidosis from Majorca focus. Amyloid 2019;26:18–19. 31343328 10.1080/13506129.2019.1582487

[60] Pomfret EA, Lewis WD, Jenkins RL, Bergethon P, Dubrey SW, Reisinger J, Falk RH, Skinner M. Effect of orthotopic liver transplantation on the progression of familial amyloidotic polyneuropathy. Transplantation 1998;65:918–925. 9565095 10.1097/00007890-199804150-00010

[61] Suhr OB, Lundgren E, Westermark P. One mutation, two distinct disease variants: unravelling the impact of transthyretin amyloid fibril composition. J Intern Med 2017;281:337–347. 28093848 10.1111/joim.12585

[62] Bergström J, Gustavsson A, Hellman U, Sletten K, Murphy CL, Weiss DT, Solomon A, Olofsson B-O, Westermark P. Amyloid deposits in transthyretin-derived amyloidosis: cleaved transthyretin is associated with distinct amyloid morphology. J Pathol 2005;206:224–232. 15810051 10.1002/path.1759

[63] Ihse E, Rapezzi C, Merlini G, Benson MD, Ando Y, Suhr OB, Ikeda S-I, Lavatelli F, Obici L, Quarta CC, Leone O, Jono H, Ueda M, Lorenzini M, Liepnieks J, Ohshima T, Tasaki M, Yamashita T, Westermark P. Amyloid fibrils containing fragmented ATTR may be the standard fibril composition in ATTR amyloidosis. Amyloid 2013;20:142–150. 23713495 10.3109/13506129.2013.797890

[64] Gustafsson S, Ihse E, Henein MY, Westermark P, Lindqvist P, Suhr OB. Amyloid fibril composition as a predictor of development of cardiomyopathy after liver transplantation for hereditary transthyretin amyloidosis. Transplantation 2012;93:1017–1023. 22395298 10.1097/TP.0b013e31824b3749

[65] Mangione PP, Porcari R, Gillmore JD, Pucci P, Monti M, Porcari M, Giorgetti S, Marchese L, Raimondi S, Serpell LC, Chen W, Relini A, Marcoux J, Clatworthy IR, Taylor GW, Tennent GA, Robinson CV, Hawkins PN, Stoppini M, Wood SP, Pepys MB, Bellotti V. Proteolytic cleavage of Ser52Pro variant transthyretin triggers its amyloid fibrillogenesis. Proc Natl Acad Sci U S A 2014;111:1539–1544. 24474780 10.1073/pnas.1317488111 PMC3910611

[66] Marcoux J, Mangione PP, Porcari R, Degiacomi MT, Verona G, Taylor GW, Giorgetti S, Raimondi S, Sanglier-Cianférani S, Benesch JLP, Cecconi C, Naqvi MM, Gillmore JD, Hawkins PN, Stoppini M, Robinson CV, Pepys MB, Bellotti V. A novel mechano-enzymatic cleavage mechanism underlies transthyretin amyloidogenesis. EMBO Mol Med 2015;7:1337–1349. 26286619 10.15252/emmm.201505357 PMC4604687

[67] Mangione PP, Verona G, Corazza A, Marcoux J, Canetti D, Giorgetti S, Raimondi S, Stoppini M, Esposito M, Relini A, Canale C, Valli M, Marchese L, Faravelli G, Obici L, Hawkins PN, Taylor GW, Gillmore JD, Pepys MB, Bellotti V. Plasminogen activation triggers transthyretin amyloidogenesis *in vitro*. J Biol Chem 2018;293:14192–14199. 30018138 10.1074/jbc.RA118.003990 PMC6139548

[68] Raimondi S, Mangione PP, Verona G, Canetti D, Nocerino P, Marchese L, Piccarducci R, Mondani V, Faravelli G, Taylor GW, Gillmore JD, Corazza A, Pepys MB, Giorgetti S, Bellotti V. Comparative study of the stabilities of synthetic *in vitro* and natural *ex vivo* transthyretin amyloid fibrils. J Biol Chem 2020;295:11379–11387. 32571879 10.1074/jbc.RA120.014026 PMC7450123

[69] Pedretti R, Wang L, Yakubovska A, Zhang QS, Nguyen B, Grodin JL, Masri A, Saelices L. Structure-based probe reveals the presence of large transthyretin aggregates in plasma of ATTR amyloidosis patients. JACC Basic Transl Sci 2024;9:1088–1100. 39444930 10.1016/j.jacbts.2024.05.013 PMC11494390

[70] Pedretti R, Wang L, Hanna M, Benson MD, Grodin JL, Tang WHW, Masri A, Saelices L. Detection of circulating transthyretin amyloid aggregates in plasma: a novel biomarker for transthyretin amyloidosis. Circulation 2024;149:1696–1699. 38768274 10.1161/CIRCULATIONAHA.123.067225 PMC11107565

[71] Liao R, Jain M, Teller P, Connors LH, Ngoy S, Skinner M, Falk RH, Apstein CS. Infusion of light chains from patients with cardiac amyloidosis causes diastolic dysfunction in isolated mouse hearts. Circulation 2001;104:1594–1597. 11581134

[72] Brenner DA, Jain M, Pimentel DR, Wang B, Connors LH, Skinner M, Apstein CS, Liao R. Human amyloidogenic light chains directly impair cardiomyocyte function through an increase in cellular oxidant stress. Circ Res 2004;94:1008–1010. 15044325 10.1161/01.RES.0000126569.75419.74

[73] Guan J, Mishra S, Qiu Y, Shi J, Trudeau K, Las G, Liesa M, Shirihai OS, Connors LH, Seldin DC, Falk RH, MacRae CA, Liao R. Lysosomal dysfunction and impaired autophagy underlie the pathogenesis of amyloidogenic light chain-mediated cardiotoxicity. EMBO Mol Med 2014;6:1493–1507. 25319546 10.15252/emmm.201404190 PMC4237473

[74] Shi J, Guan J, Jiang B, Brenner DA, Del Monte F, Ward JE, Connors LH, Sawyer DB, Semigran MJ, Macgillivray TE, Seldin DC, Falk R, Liao R. Amyloidogenic light chains induce cardiomyocyte contractile dysfunction and apoptosis via a non-canonical p38alpha MAPK pathway. Proc Natl Acad Sci U S A 2010;107:4188–4193. 20150510 10.1073/pnas.0912263107 PMC2840082

[75] Guan J, Mishra S, Shi J, Plovie E, Qiu Y, Cao X, Gianni D, Jiang B, Del Monte F, Connors LH, Seldin DC, Lavatelli F, Rognoni P, Palladini G, Merlini G, Falk RH, Semigran MJ, Dec GW, Macrae CA, Liao R. Stanniocalcin1 is a key mediator of amyloidogenic light chain induced cardiotoxicity. Basic Res Cardiol 2013;108:378. 23982491 10.1007/s00395-013-0378-5 PMC3914405

[76] Zhang Y, Yu W, Chang W, Wang M, Zhang L, Yu F. Light chain amyloidosis-induced autophagy is mediated by the Foxo3a/beclin-1 pathway in cardiomyocytes. Lab Invest 2023;103:100001. 37039144 10.1016/j.labinv.2022.100001

[77] Sikkink LA, Ramirez-Alvarado M. Cytotoxicity of amyloidogenic immunoglobulin light chains in cell culture. Cell Death Dis 2010;1:e98. 21368874 10.1038/cddis.2010.75 PMC3032327

[78] Marin-Argany M, Lin Y, Misra P, Williams A, Wall JS, Howell KG, Elsbernd LR, McClure M, Ramirez-Alvarado M. Cell damage in light chain amyloidosis: fibril internalization, toxicity and cell-mediated seeding. J Biol Chem 2016;291:19813–19825. 27462073 10.1074/jbc.M116.736736 PMC5025671

[79] Levinson RT, Olatoye OO, Randles EG, Howell KG, DiCostanzo AC, Ramirez-Alvarado M. Role of mutations in the cellular internalization of amyloidogenic light chains into cardiomyocytes. Sci Rep 2013;3:1278. 23417147 10.1038/srep01278 PMC3575045

[80] Ghosh S, Villacorta-Martin C, Lindstrom-Vautrin J, Kenney D, Golden CS, Edwards CV, Sanchorawala V, Connors LH, Giadone RM, Murphy GJ. Mapping cellular response to destabilized transthyretin reveals cell- and amyloidogenic protein-specific signatures. Amyloid 2023;30:379–393. 37439769 10.1080/13506129.2023.2224494

[81] McWilliams-Koeppen HP, Foster JS, Hackenbrack N, Ramirez-Alvarado M, Donohoe D, Williams A, Macy S, Wooliver C, Wortham D, Morrell-Falvey J, Foster CM, Kennel SJ, Wall JS. Light chain amyloid fibrils cause metabolic dysfunction in human cardiomyocytes. PLoS One 2015;10:e0137716. 26393799 10.1371/journal.pone.0137716 PMC4579077

[82] Lin Y, Marin-Argany M, Dick CJ, Redhage KR, Blancas-Mejia LM, Bulur P, Butler GW, Deeds MC, Madden BJ, Williams A, Wall JS, Dietz A, Ramirez-Alvarado M. Mesenchymal stromal cells protect human cardiomyocytes from amyloid fibril damage. Cytotherapy 2017;19:1426–1437. 29037943 10.1016/j.jcyt.2017.08.021 PMC6456258

[83] Jordan TL, Maar K, Redhage KR, Misra P, Blancas-Mejia LM, Dick CJ, Wall JS, Williams A, Dietz AB, Wijnen Av, Lin Y, Ramirez-Alvarado M. Light chain amyloidosis induced inflammatory changes in cardiomyocytes and adipose-derived mesenchymal stromal cells. Leukemia 2020;34:1383–1393. 31796914 10.1038/s41375-019-0640-4 PMC7196017

[84] Pesce M, Duda GN, Forte G, Girao H, Raya A, Roca-Cusachs P, Sluijter JPG, Tschöpe C, Van Linthout S. Cardiac fibroblasts and mechanosensation in heart development, health and disease. Nat Rev Cardiol 2023;20:309–324. 36376437 10.1038/s41569-022-00799-2

[85] Imperlini E, Gnecchi M, Rognoni P, Sabidò E, Ciuffreda MC, Palladini G, Espadas G, Mancuso FM, Bozzola M, Malpasso G, Valentini V, Palladini G, Orrù S, Ferraro G, Milani P, Perlini S, Salvatore F, Merlini G, Lavatelli F. Proteotoxicity in cardiac amyloidosis: amyloidogenic light chains affect the levels of intracellular proteins in human heart cells. Sci Rep 2017;7:15661. 29142197 10.1038/s41598-017-15424-3 PMC5688098

[86] Maritan M, Romeo M, Oberti L, Sormanni P, Tasaki M, Russo R, Ambrosetti A, Motta P, Rognoni P, Mazzini G, Barbiroli A, Palladini G, Vendruscolo M, Diomede L, Bolognesi M, Merlini G, Lavatelli F, Ricagno S. Inherent biophysical properties modulate the toxicity of soluble amyloidogenic light chains. J Mol Biol 2020;432:845–860. 31874151 10.1016/j.jmb.2019.12.015

[87] Lavatelli F, Imperlini E, Orrù S, Rognoni P, Sarnataro D, Palladini G, Malpasso G, Soriano ME, Di Fonzo A, Valentini V, Gnecchi M, Perlini S, Salvatore F, Merlini G. Novel mitochondrial protein interactors of immunoglobulin light chains causing heart amyloidosis. FASEB J 2015;29:4614–4628. 26220173 10.1096/fj.15-272179

[88] Trinkaus-Randall V, Walsh MT, Steeves S, Monis G, Connors LH, Skinner M. Cellular response of cardiac fibroblasts to amyloidogenic light chains. Am J Pathol 2005;166:197–208. 15632012 10.1016/S0002-9440(10)62244-4 PMC1602293

[89] Migrino RQ, Truran S, Gutterman DD, Franco DA, Bright M, Schlundt B, Timmons M, Motta A, Phillips SA, Hari P. Human microvascular dysfunction and apoptotic injury induced by AL amyloidosis light chain proteins. Am J Physiol Heart Circ Physiol 2011;301:H2305–H2312. 21963839 10.1152/ajpheart.00503.2011 PMC3233808

[90] Migrino RQ, Hari P, Gutterman DD, Bright M, Truran S, Schlundt B, Phillips SA. Systemic and microvascular oxidative stress induced by light chain amyloidosis. Int J Cardiol 2010;145:67–68. 19446898 10.1016/j.ijcard.2009.04.044 PMC2974792

[91] Truran S, Weissig V, Ramirez-Alvarado M, Franco DA, Burciu C, Georges J, Murarka S, Okoth WA, Schwab S, Hari P, Migrino RQ. Nanoliposomes protect against AL amyloid light chain protein-induced endothelial injury. J Liposome Res 2014;24:69–73. 24236475 10.3109/08982104.2013.838258 PMC3925072

[92] Franco DA, Truran S, Weissig V, Guzman-Villanueva D, Karamanova N, Senapati S, Burciu C, Ramirez-Alvarado M, Blancas-Mejia LM, Lindsay S, Hari P, Migrino RQ. Monosialoganglioside-containing nanoliposomes restore endothelial function impaired by AL amyloidosis light chain proteins. J Am Heart Assoc 2016;5:e003318. 27412900 10.1161/JAHA.116.003318 PMC4937272

[93] Guzman-Villanueva D, Migrino RQ, Truran S, Karamanova N, Franco DA, Burciu C, Senapati S, Nedelkov D, Hari P, Weissig V. PEGylated-nanoliposomal clusterin for amyloidogenic light chain-induced endothelial dysfunction. J Liposome Res 2018;28:97–105. 28103719 10.1080/08982104.2016.1274756 PMC5591079

[94] Zhou P, Ma X, Iyer L, Chaulagain C, Comenzo RL. One siRNA pool targeting the λ constant region stops λ light-chain production and causes terminal endoplasmic reticulum stress. Blood 2014;123:3440–3451. 24723680 10.1182/blood-2013-10-535187

[95] Arendt BK, Ramirez-Alvarado M, Sikkink LA, Keats JJ, Ahmann GJ, Dispenzieri A, Fonseca R, Ketterling RP, Knudson RA, Mulvihill EM, Tschumper RC, Wu X, Zeldenrust SR, Jelinek DF. Biologic and genetic characterization of the novel amyloidogenic lambda light chain-secreting human cell lines, ALMC-1 and ALMC-2. Blood 2008;112:1931–1941. 18567838 10.1182/blood-2008-03-143040 PMC2518895

[96] Ma X, Zhou P, Wong SW, Warner M, Chaulagain C, Comenzo RL. siRNA targeting the κ light chain constant region: preclinical testing of an approach to nonfibrillar and fibrillar light chain deposition diseases. Gene Ther 2016;23:727–733. 27383253 10.1038/gt.2016.50

[97] Hovey BM, Ward JE, Soo Hoo P, O’Hara CJ, Connors LH, Seldin DC. Preclinical development of siRNA therapeutics for AL amyloidosis. Gene Ther 2011;18:1150–1156. 21562591 10.1038/gt.2011.69 PMC3155733

[98] Renz M, Torres R, Dolan PJ, Tam SJ, Tapia JR, Li L, Salmans JR, Barbour RM, Shughrue PJ, Nijjar T, Schenk D, Kinney GG, Zago W. 2A4 binds soluble and insoluble light chain aggregates from AL amyloidosis patients and promotes clearance of amyloid deposits by phagocytosis. Amyloid 2016;23:168–177. 27494229 10.1080/13506129.2016.1205974 PMC5152553

[99] Cooley CB, Ryno LM, Plate L, Morgan GJ, Hulleman JD, Kelly JW, Wiseman RL. Unfolded protein response activation reduces secretion and extracellular aggregation of amyloidogenic immunoglobulin light chain. Proc Natl Acad Sci U S A 2014;111:13046–13051. 25157167 10.1073/pnas.1406050111 PMC4246986

[100] Monis GF, Schultz C, Ren R, Eberhard J, Costello C, Connors L, Skinner M, Trinkaus-Randall V. Role of endocytic inhibitory drugs on internalization of amyloidogenic light chains by cardiac fibroblasts. Am J Pathol 2006;169:1939–1952. 17148659 10.2353/ajpath.2006.060183 PMC1762491

[101] Berghoff M, Kathpal M, Khan F, Skinner M, Falk R, Freeman R. Endothelial dysfunction precedes C-fiber abnormalities in primary (AL) amyloidosis. Ann Neurol 2003;53:725–730. 12783418 10.1002/ana.10552

[102] Neben-Wittich MA, Wittich CM, Mueller PS, Larson DR, Gertz MA, Edwards WD. Obstructive intramural coronary amyloidosis and myocardial ischemia are common in primary amyloidosis. Am J Med 2005;118:1287. 10.1016/j.amjmed.2005.06.017 16271914

[103] Wittich CM, Neben-Wittich MA, Mueller PS, Gertz MA, Edwards WD. Deposition of amyloid proteins in the epicardial coronary arteries of 58 patients with primary systemic amyloidosis. Cardiovasc Pathol 2007;16:75–78. 17317539 10.1016/j.carpath.2006.09.011

[104] Franco DA, Truran S, Burciu C, Gutterman DD, Maltagliati A, Weissig V, Hari P, Migrino RQ. Protective role of clusterin in preserving endothelial function in AL amyloidosis. Atherosclerosis 2012;225:220–223. 22981431 10.1016/j.atherosclerosis.2012.08.028 PMC3478430

[105] Meng X, Fink AL, Uversky VN. The effect of membranes on the *in vitro* fibrillation of an amyloidogenic light-chain variable-domain SMA. J Mol Biol 2008;381:989–999. 18619464 10.1016/j.jmb.2008.06.062 PMC2556633

[106] Gobbi M, Re F, Canovi M, Beeg M, Gregori M, Sesana S, Sonnino S, Brogioli D, Musicanti C, Gasco P, Salmona M, Masserini ME. Lipid-based nanoparticles with high binding affinity for amyloid-beta1-42 peptide. Biomaterials 2010;31:6519–6529. 20553982 10.1016/j.biomaterials.2010.04.044

[107] Re F, Cambianica I, Sesana S, Salvati E, Cagnotto A, Salmona M, Couraud P-O, Moghimi SM, Masserini M, Sancini G. Functionalization with ApoE-derived peptides enhances the interaction with brain capillary endothelial cells of nanoliposomes binding amyloid-beta peptide. J Biotechnol 2011;156:341–346. 21763360 10.1016/j.jbiotec.2011.06.037

[108] Truran S, Weissig V, Madine J, Davies HA, Guzman-Villanueva D, Franco DA, Karamanova N, Burciu C, Serrano G, Beach TG, Migrino RQ. Nanoliposomes protect against human arteriole endothelial dysfunction induced by β-amyloid peptide. J Cereb Blood Flow Metab 2016;36:405–412. 26661197 10.1177/0271678X15610134 PMC4759678

[109] Palladini G, Lavatelli F, Russo P, Perlini S, Perfetti V, Bosoni T, Obici L, Bradwell AR, D’Eril GM, Fogari R, Moratti R, Merlini G. Circulating amyloidogenic free light chains and serum N-terminal natriuretic peptide type B decrease simultaneously in association with improvement of survival in AL. Blood 2006;107:3854–3858. 16434487 10.1182/blood-2005-11-4385

[110] Gertz MA . Immunoglobulin light chain amyloidosis: 2024 update on diagnosis, prognosis, and treatment. Am J Hematol 2024;99:309–324. 38095141 10.1002/ajh.27177

[111] Mahadevia H, Ponvilawan B, Sharma P, Al-Obaidi A, Qasim H, Koyi J, Anwer F, Raza S. Advancements and future trends of immunotherapy in light-chain amyloidosis. Crit Rev Oncol Hematol 2023;183:103917. 36696931 10.1016/j.critrevonc.2023.103917

[112] Wall JS, Kennel SJ, Williams A, Richey T, Stuckey A, Huang Y, Macy S, Donnell R, Barbour R, Seubert P, Schenk D. AL amyloid imaging and therapy with a monoclonal antibody to a cryptic epitope on amyloid fibrils. PLoS One 2012;7:e52686. 23300743 10.1371/journal.pone.0052686 PMC3530443

[113] Gertz MA, Cohen AD, Comenzo RL, Kastritis E, Landau HJ, Libby EN, Liedtke M, Sanchorawala V, Schönland S, Wechalekar A, Zonder JA, Palladini G, Walling J, Guthrie S, Nie C, Karp C, Jin Y, Kinney GG, Merlini G. Birtamimab plus standard of care in light-chain amyloidosis: the phase 3 randomized placebo-controlled VITAL trial. Blood 2023;142:1208–1218. 37366170 10.1182/blood.2022019406 PMC10644097

[114] Emdin M, Morfino P, Crosta L, Aimo A, Vergaro G, Castiglione V. Monoclonal antibodies and amyloid removal as a therapeutic strategy for cardiac amyloidosis. Eur Heart J Suppl 2023;25:B79–B84. 37091656 10.1093/eurheartjsupp/suad079 PMC10120953

[115] Edwards CV, Rao N, Bhutani D, Mapara M, Radhakrishnan J, Shames S, Maurer MS, Leng S, Solomon A, Lentzsch S, Eisenberger A. Phase 1a/b study of monoclonal antibody CAEL-101 (11-1F4) in patients with AL amyloidosis. Blood 2021;138:2632–2641. 34521113 10.1182/blood.2020009039 PMC8703360

[116] Benian GM, Epstein HF. *Caenorhabditis elegans* muscle: a genetic and molecular model for protein interactions in the heart. Circ Res 2011;109:1082–1095. 21998299 10.1161/CIRCRESAHA.110.237685

[117] Romeo M, Barzago MM, Corbelli A, Maglioni S, Ventura N, Natale C, Conz A, Salmona M, Palladini G, Nuvolone M, Fiordaliso F, Merlini G, Diomede L. Modeling immunoglobulin light chain amyloidosis in *Caenorhabditis elegans*. Dis Model Mech 2025;18:dmm052230. 40709584 10.1242/dmm.052230 PMC12320968

[118] Diomede L, Rognoni P, Lavatelli F, Romeo M, del Favero E, Cantù L, Ghibaudi E, di Fonzo A, Corbelli A, Fiordaliso F, Palladini G, Valentini V, Perfetti V, Salmona M, Merlini G. A *Caenorhabditis elegans*-based assay recognizes immunoglobulin light chains causing heart amyloidosis. Blood 2014;123:3543–3552. 24665135 10.1182/blood-2013-10-525634 PMC4047494

[119] Diomede L, Romeo M, Rognoni P, Beeg M, Foray C, Ghibaudi E, Palladini G, Cherny RA, Verga L, Capello GL, Perfetti V, Fiordaliso F, Merlini G, Salmona M. Cardiac light chain amyloidosis: the role of metal ions in oxidative stress and mitochondrial damage. Antioxid Redox Signal 2017;27:567–582. 28132512 10.1089/ars.2016.6848 PMC5567464

[120] Russo R, Romeo M, Schulte T, Maritan M, Oberti L, Barzago MM, Barbiroli A, Pappone C, Anastasia L, Palladini G, Diomede L, Ricagno S. Cu(ii) binding increases the soluble toxicity of amyloidogenic light chains. Int J Mol Sci 2022;23:950. 35055136 10.3390/ijms23020950 PMC8780072

[121] Mishra S, Joshi S, Ward JE, Buys EP, Mishra D, Mishra D, Morgado I, Fisch S, Lavatelli F, Merlini G, Dorbala S, MacRae CA, Liao R. Zebrafish model of amyloid light chain cardiotoxicity: regeneration versus degeneration. Am J Physiol Heart Circ Physiol 2019;316:H1158–H1166. 30875258 10.1152/ajpheart.00788.2018 PMC6580397

[122] Dispenzieri A, Lacy MQ, Katzmann JA, Rajkumar SV, Abraham RS, Hayman SR, Kumar SK, Clark R, Kyle RA, Litzow MR, Inwards DJ, Ansell SM, Micallef IM, Porrata LF, Elliott MA, Johnston PB, Greipp PR, Witzig TE, Zeldenrust SR, Russell SJ, Gastineau D, Gertz MA. Absolute values of immunoglobulin free light chains are prognostic in patients with primary systemic amyloidosis undergoing peripheral blood stem cell transplantation. Blood 2006;107:3378–3383. 16397135 10.1182/blood-2005-07-2922 PMC1895763

[123] Mishra S, Guan J, Plovie E, Seldin DC, Connors LH, Merlini G, Falk RH, MacRae CA, Liao R. Human amyloidogenic light chain proteins result in cardiac dysfunction, cell death, and early mortality in zebrafish. Am J Physiol Heart Circ Physiol 2013;305:H95–H103. 23624626 10.1152/ajpheart.00186.2013 PMC3727100

[124] Bournele D, Beis D. Zebrafish models of cardiovascular disease. Heart Fail Rev 2016;21:803–813. 27503203 10.1007/s10741-016-9579-y

[125] Bakkers J . Zebrafish as a model to study cardiac development and human cardiac disease. Cardiovasc Res 2011;91:279–288. 21602174 10.1093/cvr/cvr098 PMC3125074

[126] Guan J, Mishra S, Qiu Y, Shi J, Trudeau K, Las G, Liesa M, Shirihai OS, Connors LH, Seldin DC, Falk RH, MacRae CA, Liao R. Lysosomal dysfunction and impaired autophagy underlie the pathogenesis of amyloidogenic light chain-mediated cardiotoxicity. EMBO Mol Med 2015;7:688. 25940533 10.15252/emmm.201505318 PMC4492824

[127] Shin JT, Ward JE, Collins PA, Dai M, Semigran HL, Semigran MJ, Seldin DC. Overexpression of human amyloidogenic light chains causes heart failure in embryonic zebrafish: a preliminary report. Amyloid 2012;19:191–196. 23126591 10.3109/13506129.2012.733741

[128] Solomon A, Weiss DT, Pepys MB. Induction in mice of human light-chain-associated amyloidosis. Am J Pathol 1992;140:629–637. 1546744 PMC1886151

[129] Hrncic R, Wall J, Wolfenbarger DA, Murphy CL, Schell M, Weiss DT, Solomon A. Antibody-mediated resolution of light chain-associated amyloid deposits. Am J Pathol 2000;157:1239–1246. 11021828 10.1016/S0002-9440(10)64639-1 PMC1850152

[130] Richey T, Foster JS, Williams AD, Williams AB, Stroh A, Macy S, Wooliver C, Heidel RE, Varanasi SK, Ergen EN, Trent DJ, Kania SA, Kennel SJ, Martin EB, Wall JS. Macrophage-mediated phagocytosis and dissolution of amyloid-like fibrils in mice, monitored by optical imaging. Am J Pathol 2019;189:989–998. 30735627 10.1016/j.ajpath.2019.01.011 PMC6521888

[131] Beierle SP, Foster JS, Richey T, Stuckey A, Macy S, Kennel SJ, Wall JS. A novel murine system for validating the specific targeting of peptides to light chain associated (AL) amyloid. Amyloid 2017;24:74–75. 28434353 10.1080/13506129.2017.1295377 PMC6355328

[132] Solomon A, Weiss DT, Wall JS. Therapeutic potential of chimeric amyloid-reactive monoclonal antibody 11-1F4. Clin Cancer Res 2003;9:3831S–3838S. 14506180

[133] Wall JS, Kennel SJ, Paulus M, Gregor J, Richey T, Avenell J, Yap J, Townsend D, Weiss DT, Solomon A. Radioimaging of light chain amyloid with a fibril-reactive monoclonal antibody. J Nucl Med 2006;47:2016–2024. 17138745 PMC1866271

[134] Wall JS, Martin EB, Endsley A, Stuckey AC, Williams AD, Powell D, Whittle B, Hall S, Lambeth TR, Julian RR, Stabin M, Lands RH, Kennel SJ. First in human evaluation and dosimetry calculations for peptide 124I-p5 + 14-a novel radiotracer for the detection of systemic amyloidosis using PET/CT imaging. Mol Imaging Biol 2022;24:479–488. 34786667 10.1007/s11307-021-01681-2

[135] Gertz MA, Landau H, Comenzo RL, Seldin D, Weiss B, Zonder J, Merlini G, Schönland S, Walling J, Kinney GG, Koller M, Schenk DB, Guthrie SD, Liedtke M. First-in-human phase I/II study of NEOD001 in patients with light chain amyloidosis and persistent organ dysfunction. J Clin Oncol 2016;34:1097–1103. 26858336 10.1200/JCO.2015.63.6530 PMC5470113

[136] Gertz MA, Landau HJ, Weiss BM. Organ response in patients with AL amyloidosis treated with NEOD001, an amyloid-directed monoclonal antibody. Am J Hematol 2016;91:E506–E508. 27648922 10.1002/ajh.24563 PMC5132098

[137] Edwards CV, Gould J, Langer AL, Mapara M, Radhakrishnan J, Maurer MS, Raza S, Mears JG, Wall J, Solomon A, Lentzsch S. Interim analysis of the phase 1a/b study of chimeric fibril-reactive monoclonal antibody 11-1F4 in patients with AL amyloidosis. Amyloid 2017;24:58–59. 28434347 10.1080/13506129.2017.1292900

[138] Foster JS, Balachandran M, Hancock TJ, Martin EB, Macy S, Wooliver C, Richey T, Stuckey A, Williams AD, Jackson JW, Kennel SJ, Wall JS. Development and characterization of a prototypic pan-amyloid clearing agent—a novel murine peptide-immunoglobulin fusion. Front Immunol 2023;14:1275372. 37854603 10.3389/fimmu.2023.1275372 PMC10580800

[139] Ward JE, SooHoo P, Toraldo G, Jasuja R, Connors LH, O’Hara C, Seldin DC. Metabolic phenotype in an AL amyloidosis transgenic mouse model. Amyloid 2011;18:40–41. 21838426 10.3109/13506129.2011.574354014 PMC5603191

[140] Nuvolone M, Sorce S, Pelczar P, Rushing E, Lavatelli F, Rognoni P, Valentini V, Palladini G, Merlini G, Aguzzi A. Regulated expression of amyloidogenic immunoglobulin light chains in mice. Amyloid 2017;24:52–53. 10.1080/13506129.2017.1289914 28434289

[141] Martinez-Rivas G, Ayala MV, Bender S, Codo GR, Swiderska K, Lampis A, Pedroza L, Merdanovic M, Sicard P, Pinault E, Richard L, Lavatelli F, Giorgetti S, Canetti D, Rinsant A, Kaaki S, Ory C, Oblet C, Pollet J, Naser E, Carpinteiro A, Roussel M, Javaugue V, Jaccard A, Bonaud A, Delpy L, Ehrmann M, Bridoux F, Sirac C. A mouse model of cardiac AL amyloidosis unveils mechanisms of tissue accumulation and toxicity of amyloid fibrils. BioRxiv. 2024. doi:10.1038/s41467-025-58307-2 PMC11950232 40148271

[142] Ayala MV, Bender S, Anegon I, Menoret S, Bridoux F, Jaccard A, Sirac C. A rat model expressing a human amyloidogenic kappa light chain. Amyloid 2021;28:209–210. 33480298 10.1080/13506129.2021.1877651

[143] Bourgault S, Choi S, Buxbaum JN, Kelly JW, Price JL, Reixach N. Mechanisms of transthyretin cardiomyocyte toxicity inhibition by resveratrol analogs. Biochem Biophys Res Commun 2011;410:707–713. 21557933 10.1016/j.bbrc.2011.04.133 PMC3145458

[144] Manral P, Reixach N. Amyloidogenic and non-amyloidogenic transthyretin variants interact differently with human cardiomyocytes: insights into early events of non-fibrillar tissue damage. Biosci Rep 2015;35:e00172 25395306 10.1042/BSR20140155 PMC4293901

[145] Penchala SC, Connelly S, Wang Y, Park MS, Zhao L, Baranczak A, Rappley I, Vogel H, Liedtke M, Witteles RM, Powers ET, Reixach N, Chan WK, Wilson IA, Kelly JW, Graef IA, Alhamadsheh MM. AG10 inhibits amyloidogenesis and cellular toxicity of the familial amyloid cardiomyopathy-associated V122I transthyretin. Proc Natl Acad Sci U S A 2013;110:9992–9997. 23716704 10.1073/pnas.1300761110 PMC3683741

[146] Sant’Anna R, Gallego P, Robinson LZ, Pereira-Henriques A, Ferreira N, Pinheiro F, Esperante S, Pallares I, Huertas O, Almeida MR, Reixach N, Insa R, Velazquez-Campoy A, Reverter D, Reig N, Ventura S. Repositioning tolcapone as a potent inhibitor of transthyretin amyloidogenesis and associated cellular toxicity. Nat Commun 2016;7:10787. 26902880 10.1038/ncomms10787 PMC4766415

[147] Leri M, Rebuzzini P, Caselli A, Luti S, Natalello A, Giorgetti S, Marchese L, Garagna S, Stefani M, Paoli P, Bucciantini M. S-Homocysteinylation effects on transthyretin: worsening of cardiomyopathy onset. Biochim Biophys Acta Gen Subj 2020;1864:129453. 31676294 10.1016/j.bbagen.2019.129453

[148] Sartiani L, Bucciantini M, Spinelli V, Leri M, Natalello A, Nosi D, Maria Doglia S, Relini A, Penco A, Giorgetti S, Gerace E, Mannaioni G, Bellotti V, Rigacci S, Cerbai E, Stefani M. Biochemical and electrophysiological modification of amyloid transthyretin on cardiomyocytes. Biophys J 2016;111:2024–2038. 27806283 10.1016/j.bpj.2016.09.010 PMC5103001

[149] Ami D, Mereghetti P, Leri M, Giorgetti S, Natalello A, Doglia SM, Stefani M, Bucciantini M. A FTIR microspectroscopy study of the structural and biochemical perturbations induced by natively folded and aggregated transthyretin in HL-1 cardiomyocytes. Sci Rep 2018;8:12508. 30131519 10.1038/s41598-018-30995-5 PMC6104026

[150] George J, Rappaport M, Shimoni S, Goland S, Voldarsky I, Fabricant Y, Edri O, Cuciuc V, Lifshitz S, Tshori S, Fassler M. A novel monoclonal antibody targeting aggregated transthyretin facilitates its removal and functional recovery in an experimental model. Eur Heart J 2020;41:1260–1270. 31865366 10.1093/eurheartj/ehz695

[151] Dittloff KT, Spanghero E, Solís C, Banach K, Russell B. Transthyretin deposition alters cardiomyocyte sarcomeric architecture, calcium transients, and contractile force. Physiol Rep 2022;10:e15207. 35262277 10.14814/phy2.15207 PMC8906053

[152] Hein S, Furkel J, Knoll M, Aus dem Siepen F, Schönland S, Hegenbart U, Katus HA, Kristen AV, Konstandin MH. Impaired *in vitro* growth response of plasma-treated cardiomyocytes predicts poor outcome in patients with transthyretin amyloidosis. Clin Res Cardiol 2021;110:579–590. 33481097 10.1007/s00392-020-01801-y PMC8055573

[153] Leung A, Nah SK, Reid W, Ebata A, Koch CM, Monti S, Genereux JC, Wiseman RL, Wolozin B, Connors LH, Berk JL, Seldin DC, Mostoslavsky G, Kotton DN, Murphy GJ. Induced pluripotent stem cell modeling of multisystemic, hereditary transthyretin amyloidosis. Stem Cell Reports 2013;1:451–463. 24286032 10.1016/j.stemcr.2013.10.003 PMC3841264

[154] Giadone RM, Rosarda JD, Akepati PR, Thomas AC, Boldbaatar B, James MF, Wilson AA, Sanchorawala V, Connors LH, Berk JL, Wiseman RL, Murphy GJ. A library of ATTR amyloidosis patient-specific induced pluripotent stem cells for disease modelling and *in vitro* testing of novel therapeutics. Amyloid 2018;25:148–155. 30032658 10.1080/13506129.2018.1489228 PMC6319917

[155] Bonilauri B, Shin HS, Htet M, Yan CD, Witteles RM, Sallam K, Wu JC. Generation of two induced pluripotent stem cell lines from patients with cardiac amyloidosis carrying heterozygous transthyretin (TTR) mutation. Stem Cell Res 2023;72:103215. 37788558 10.1016/j.scr.2023.103215 PMC10821799

[156] Ouchi K, Isono K, Ohya Y, Shiraki N, Tasaki M, Inomata Y, Ueda M, Era T, Kume S, Ando Y, Jono H. Characterization of heterozygous ATTR Tyr114Cys amyloidosis-specific induced pluripotent stem cells. Heliyon 2024;10:e24590. 38312695 10.1016/j.heliyon.2024.e24590 PMC10835262

[157] Misumi Y, Ando Y, Gonçalves NP, Saraiva MJ. Fibroblasts endocytose and degrade transthyretin aggregates in transthyretin-related amyloidosis. Lab Invest 2013;93:911–920. 23817086 10.1038/labinvest.2013.83

[158] Dittloff KT, Iezzi A, Zhong JX, Mohindra P, Desai TA, Russell B. Transthyretin amyloid fibrils alter primary fibroblast structure, function, and inflammatory gene expression. Am J Physiol Heart Circ Physiol 2021;321:H149–H160. 34018852 10.1152/ajpheart.00073.2021 PMC8321815

[159] Nunes RJ, Oliveira Pd, Lages A, Becker JD, Marcelino P, Barroso E, Perdigoto R, Kelly JW, Quintas A, Santos SCR. Transthyretin proteins regulate angiogenesis by conferring different molecular identities to endothelial cells. J Biol Chem 2013;288:31752–31760. 24030829 10.1074/jbc.M113.469858 PMC3814769

[160] Ministrini S, Niederberger R, Akhmedov A, Beer G, Puspitasari YM, Franzini M, Vergaro G, Cannie DE, Elliott P, Kahr PC, Hock C, Kobza R, Toggweiler S, Lüscher TF, Camici GG, Stämpfli SF. Antithrombotic properties of Tafamidis: an additional protective effect for transthyretin amyloid cardiomyopathy patients. Vascul Pharmacol 2024;156:107411. 39029855 10.1016/j.vph.2024.107411

[161] Sato T, Susuki S, Suico MA, Miyata M, Ando Y, Mizuguchi M, Takeuchi M, Dobashi M, Shuto T, Kai H. Endoplasmic reticulum quality control regulates the fate of transthyretin variants in the cell. EMBO J 2007;26:2501–2512. 17431395 10.1038/sj.emboj.7601685 PMC1868898

[162] Chen JJ, Genereux JC, Qu S, Hulleman JD, Shoulders MD, Wiseman RL. ATF6 activation reduces the secretion and extracellular aggregation of destabilized variants of an amyloidogenic protein. Chem Biol 2014;21:1564–1574. 25444553 10.1016/j.chembiol.2014.09.009 PMC4254654

[163] Shoulders MD, Ryno LM, Genereux JC, Moresco JJ, Tu PG, Wu C, Yates JR, Su AI, Kelly JW, Wiseman RL. Stress-independent activation of XBP1s and/or ATF6 reveals three functionally diverse ER proteostasis environments. Cell Rep 2013;3:1279–1292. 23583182 10.1016/j.celrep.2013.03.024 PMC3754422

[164] Mesgarzadeh JS, Romine IC, Smith-Cohen EM, Grandjean JMD, Kelly JW, Genereux JC, Wiseman RL. ATF6 activation reduces amyloidogenic transthyretin secretion through increased interactions with endoplasmic reticulum proteostasis factors. Cells 2022;11:1661. 35626697 10.3390/cells11101661 PMC9139617

[165] Chacko L, Kotecha T, Ioannou A, Patel N, Martinez-Naharro A, Razvi Y, Patel R, Massa P, Venneri L, Brown J, Porcari A, Knott K, Manisty C, Knight D, Lockie T, Rakhit R, Lachmann H, Wechelakar A, Whelan C, Ponticos M, Moon J, González A, Gilbertson J, Riefolo M, Leone O, Xue H, Hawkins P, Kellman P, Gillmore J, Fontana M. Myocardial perfusion in cardiac amyloidosis. Eur J Heart Fail 2024;26:598–609. 38247182 10.1002/ejhf.3137

[166] Larsen BT, Mereuta OM, Dasari S, Fayyaz AU, Theis JD, Vrana JA, Grogan M, Dogan A, Dispenzieri A, Edwards WD, Kurtin PJ, Maleszewski JJ. Correlation of histomorphological pattern of cardiac amyloid deposition with amyloid type: a histological and proteomic analysis of 108 cases. Histopathology 2016;68:648–656. 26212778 10.1111/his.12793

[167] Alencar Neto AC, Cafezeiro CRF, Bueno BVK, Ribeiro De Souza F, Henrique Rissato JHS, Souza Borges T, Freitas Carvalhal S, Santos Lima M, Alberto Buchpiguel C, Azem Chalela W, Alvarez Ramires FJ, Shcolnik Szor R, Kalil Filho R, Rochitte CE, Fernandes F. Coronary flow reserve by PET 13N-ammonia in patients with hereditary transthyretin amyloidosis with and without cardiac involvement. Eur Heart J 2022;43:ehac544.1778.

[168] Braakman I, Bulleid NJ. Protein folding and modification in the mammalian endoplasmic reticulum. Annu Rev Biochem 2011;80:71–99. 21495850 10.1146/annurev-biochem-062209-093836

[169] Sekijima Y, Wiseman RL, Matteson J, Hammarström P, Miller SR, Sawkar AR, Balch WE, Kelly JW. The biological and chemical basis for tissue-selective amyloid disease. Cell 2005;121:73–85. 15820680 10.1016/j.cell.2005.01.018

[170] Giadone RM, Liberti DC, Matte TM, Rosarda JD, Torres-Arancivia C, Ghosh S, Diedrich JK, Pankow S, Skvir N, Jean JC, Yates JR, Wilson AA, Connors LH, Kotton DN, Wiseman RL, Murphy GJ. Expression of amyloidogenic transthyretin drives hepatic proteostasis remodeling in an induced pluripotent stem cell model of systemic amyloid disease. Stem Cell Reports 2020;15:515–528. 32735824 10.1016/j.stemcr.2020.07.003 PMC7419739

[171] Ren J, Bi Y, Sowers JR, Hetz C, Zhang Y. Endoplasmic reticulum stress and unfolded protein response in cardiovascular diseases. Nat Rev Cardiol 2021;18:499–521. 33619348 10.1038/s41569-021-00511-w

[172] Madhivanan K, Greiner ER, Alves-Ferreira M, Soriano-Castell D, Rouzbeh N, Aguirre CA, Paulsson JF, Chapman J, Jiang X, Ooi FK, Lemos C, Dillin A, Prahlad V, Kelly JW, Encalada SE. Cellular clearance of circulating transthyretin decreases cell-nonautonomous proteotoxicity in *Caenorhabditis elegans*. Proc Natl Acad Sci U S A 2018;115:E7710–E7719. 30061394 10.1073/pnas.1801117115 PMC6099907

[173] Teng MH, Yin JY, Vidal R, Ghiso J, Kumar A, Rabenou R, Shah A, Jacobson DR, Tagoe C, Gallo G, Buxbaum J. Amyloid and nonfibrillar deposits in mice transgenic for wild-type human transthyretin: a possible model for senile systemic amyloidosis. Lab Invest 2001;81:385–396. 11310831 10.1038/labinvest.3780246

[174] Buxbaum JN, Tagoe C, Gallo G, Walker JR, Kurian S, Salomon DR. Why are some amyloidoses systemic? Does hepatic “chaperoning at a distance” prevent cardiac deposition in a transgenic model of human senile systemic (transthyretin) amyloidosis? FASEB J 2012;26:2283–2293. 22362898 10.1096/fj.11-189571 PMC3360152

[175] Sousa MM, Fernandes R, Palha JA, Taboada A, Vieira P, Saraiva MJ. Evidence for early cytotoxic aggregates in transgenic mice for human transthyretin Leu55Pro. Am J Pathol 2002;161:1935–1948. 12414539 10.1016/S0002-9440(10)64469-0 PMC1850789

[176] Santos SD, Fernandes R, Saraiva MJ. The heat shock response modulates transthyretin deposition in the peripheral and autonomic nervous systems. Neurobiol Aging 2010;31:280–289. 18485534 10.1016/j.neurobiolaging.2008.04.001

[177] Teixeira C, Martins HS, Saraiva MJ. Cellular environment of TTR deposits in an animal model of ATTR-cardiomyopathy. Front Mol Biosci 2023;10:1144049. 36968272 10.3389/fmolb.2023.1144049 PMC10030511

[178] Slamova I, Adib R, Ellmerich S, Golos MR, Gilbertson JA, Botcher N, Canetti D, Taylor GW, Rendell N, Tennent GA, Verona G, Porcari R, Mangione PP, Gillmore JD, Pepys MB, Bellotti V, Hawkins PN, Al-Shawi R, Simons JP. Plasmin activity promotes amyloid deposition in a transgenic model of human transthyretin amyloidosis. Nat Commun 2021;12:7112. 34876572 10.1038/s41467-021-27416-z PMC8651690

[179] Michalon A, Hagenbuch A, Huy C, Varela E, Combaluzier B, Damy T, Suhr OB, Saraiva MJ, Hock C, Nitsch RM, Grimm J. A human antibody selective for transthyretin amyloid removes cardiac amyloid through phagocytic immune cells. Nat Commun 2021;12:3142. 34035264 10.1038/s41467-021-23274-x PMC8149704

[180] Reixach N, Foss TR, Santelli E, Pascual J, Kelly JW, Buxbaum JN. Human-murine transthyretin heterotetramers are kinetically stable and non-amyloidogenic. A lesson in the generation of transgenic models of diseases involving oligomeric proteins. J Biol Chem 2008;283:2098–2107. 18006495 10.1074/jbc.M708028200

[181] Cardoso I, Martins D, Ribeiro T, Merlini G, Saraiva MJ. Synergy of combined doxycycline/TUDCA treatment in lowering transthyretin deposition and associated biomarkers: studies in FAP mouse models. J Transl Med 2010;8:74. 20673327 10.1186/1479-5876-8-74 PMC2922089

[182] Ferreira N, Pereira-Henriques A, Attar A, Klärner F-G, Schrader T, Bitan G, Gales L, Saraiva MJ, Almeida MR. Molecular tweezers targeting transthyretin amyloidosis. Neurotherapeutics 2014;11:450–461. 24459092 10.1007/s13311-013-0256-8 PMC3996111

[183] Ferreira N, Gonçalves NP, Saraiva MJ, Almeida MR. Curcumin: a multi-target disease-modifying agent for late-stage transthyretin amyloidosis. Sci Rep 2016;6:26623. 27197872 10.1038/srep26623 PMC4873750

[184] Butler JS, Chan A, Costelha S, Fishman S, Willoughby JLS, Borland TD, Milstein S, Foster DJ, Gonçalves P, Chen Q, Qin J, Bettencourt BR, Sah DW, Alvarez R, Rajeev KG, Manoharan M, Fitzgerald K, Meyers RE, Nochur SV, Saraiva MJ, Zimmermann TS. Preclinical evaluation of RNAi as a treatment for transthyretin-mediated amyloidosis. Amyloid 2016;23:109–118. 27033334 10.3109/13506129.2016.1160882 PMC4898164

[185] Li X, Lyu Y, Shen J, Mu Y, Qiang L, Liu L, Araki K, Imbimbo BP, Yamamura K-I, Jin S, Li Z. Amyloid deposition in a mouse model humanized at the transthyretin and retinol-binding protein 4 loci. Lab Invest 2018;98:512–524. 29330472 10.1038/s41374-017-0019-y

[186] Finn JD, Smith AR, Patel MC, Shaw L, Youniss MR, Heteren Jv, Dirstine T, Ciullo C, Lescarbeau R, Seitzer J, Shah RR, Shah A, Ling D, Growe J, Pink M, Rohde E, Wood KM, Salomon WE, Harrington WF, Dombrowski C, Strapps WR, Chang Y, Morrissey DV. A single administration of crispr/cas9 lipid nanoparticles achieves robust and persistent *in vivo* genome editing. Cell Rep 2018;22:2227–2235. 29490262 10.1016/j.celrep.2018.02.014

[187] Gillmore JD, Gane E, Taubel J, Kao J, Fontana M, Maitland ML, Seitzer J, O’Connell D, Walsh KR, Wood K, Phillips J, Xu Y, Amaral A, Boyd AP, Cehelsky JE, McKee MD, Schiermeier A, Harari O, Murphy A, Kyratsous CA, Zambrowicz B, Soltys R, Gutstein DE, Leonard J, Sepp-Lorenzino L, Lebwohl D. CRISPR-Cas9 in vivo gene editing for transthyretin amyloidosis. N Engl J Med 2021;385:493–502. 34215024 10.1056/NEJMoa2107454

[188] Chambers JK, Kanda T, Shirai A, Higuchi K, Ikeda S-I, Une Y. Senile systemic amyloidosis in an aged savannah monkey (*Cercopithecus aethiops*) with tenosynovial degeneration. J Vet Med Sci 2010;72:657–659. 20075604 10.1292/jvms.09-0394

[189] Nakamura S, Okabayashi S, Ageyama N, Koie H, Sankai T, Ono F, Fujimoto K, Terao K. Transthyretin amyloidosis and two other aging-related amyloidoses in an aged vervet monkey. Vet Pathol 2008;45:67–72. 18192580 10.1354/vp.45-1-67

[190] Ueda M, Ageyama N, Nakamura S, Nakamura M, Chambers JK, Misumi Y, Mizuguchi M, Shinriki S, Kawahara S, Tasaki M, Jono H, Obayashi K, Sasaki E, Une Y, Ando Y. Aged vervet monkeys developing transthyretin amyloidosis with the human disease-causing Ile122 allele: a valid pathological model of the human disease. Lab Invest 2012;92:474–484. 22184092 10.1038/labinvest.2011.195

[191] Iadanza MG, Jackson MP, Hewitt EW, Ranson NA, Radford SE. A new era for understanding amyloid structures and disease. Nat Rev Mol Cell Biol 2018;19:755–773. 30237470 10.1038/s41580-018-0060-8 PMC7617691

[192] Klimtchuk ES, Prokaeva TB, Spencer BH, Gursky O, Connors LH. *In vitro* co-expression of human amyloidogenic immunoglobulin light and heavy chain proteins: a relevant cell-based model of AL amyloidosis. Amyloid 2017;24:115–122. 10.1080/13506129.2017.1336996 PMC5580339 28632419

[193] Reixach N, Deechongkit S, Jiang X, Kelly JW, Buxbaum JN. Tissue damage in the amyloidoses: transthyretin monomers and nonnative oligomers are the major cytotoxic species in tissue culture. Proc Natl Acad Sci U S A 2004;101:2817–2822. 14981241 10.1073/pnas.0400062101 PMC365703

[194] Schmidt M, Wiese S, Adak V, Engler J, Agarwal S, Fritz G, Westermark P, Zacharias M, Fändrich M. Cryo-EM structure of a transthyretin-derived amyloid fibril from a patient with hereditary ATTR amyloidosis. Nat Commun 2019;10:5008. 31676763 10.1038/s41467-019-13038-z PMC6825171

[195] Radamaker L, Baur J, Huhn S, Haupt C, Hegenbart U, Schönland S, Bansal A, Schmidt M, Fändrich M. Cryo-EM reveals structural breaks in a patient-derived amyloid fibril from systemic AL amyloidosis. Nat Commun 2021;12:875. 33558536 10.1038/s41467-021-21126-2 PMC7870857

[196] Radamaker L, Karimi-Farsijani S, Andreotti G, Baur J, Neumann M, Schreiner S, Berghaus N, Motika R, Haupt C, Walther P, Schmidt V, Huhn S, Hegenbart U, Schönland SO, Wiese S, Read C, Schmidt M, Fändrich M. Role of mutations and post-translational modifications in systemic AL amyloidosis studied by cryo-EM. Nat Commun 2021;12:6434. 34741031 10.1038/s41467-021-26553-9 PMC8571268

[197] Richards DJ, Li Y, Kerr CM, Yao J, Beeson GC, Coyle RC, Chen X, Jia J, Damon B, Wilson R, Starr Hazard E, Hardiman G, Menick DR, Beeson CC, Yao H, Ye T, Mei Y. Human cardiac organoids for the modelling of myocardial infarction and drug cardiotoxicity. Nat Biomed Eng 2020;4:446–462. 32284552 10.1038/s41551-020-0539-4 PMC7422941

[198] Weinberger F, Mannhardt I, Eschenhagen T. Engineering cardiac muscle tissue: a maturating field of research. Circ Res 2017;120:1487–1500. 28450366 10.1161/CIRCRESAHA.117.310738

[199] Goldfracht I, Efraim Y, Shinnawi R, Kovalev E, Huber I, Gepstein A, Arbel G, Shaheen N, Tiburcy M, Zimmermann WH, Machluf M, Gepstein L. Engineered heart tissue models from hiPSC-derived cardiomyocytes and cardiac ECM for disease modeling and drug testing applications. Acta Biomater 2019;92:145–159. 31075518 10.1016/j.actbio.2019.05.016

[200] Perbellini F, Thum T. Living myocardial slices: a novel multicellular model for cardiac translational research. Eur Heart J 2020;41:2405–2408. 31711161 10.1093/eurheartj/ehz779 PMC7327529

[201] Pitoulis FG, Nunez-Toldra R, Xiao K, Kit-Anan W, Mitzka S, Jabbour RJ, Harding SE, Perbellini F, Thum T, Tombe Pd, Terracciano CM. Remodelling of adult cardiac tissue subjected to physiological and pathological mechanical load *in vitro*. Cardiovasc Res 2022;118:814–827. 33723566 10.1093/cvr/cvab084 PMC8859636

[202] Nunez-Toldra R, Kirwin T, Ferraro E, Pitoulis FG, Nicastro L, Bardi I, Kit-Anan W, Gorelik J, Simon AR, Terracciano CM. Mechanosensitive molecular mechanisms of myocardial fibrosis in living myocardial slices. ESC Heart Fail 2022;9:1400–1412. 35128823 10.1002/ehf2.13832 PMC8934971

[203] Paloschi V, Sabater-Lleal M, Middelkamp H, Vivas A, Johansson S, van der Meer A, Tenje M, Maegdefessel L. Organ-on-a-chip technology: a novel approach to investigate cardiovascular diseases. Cardiovasc Res 2021;117:2742–2754. 33729461 10.1093/cvr/cvab088 PMC8683705

[204] Bourgault S, Solomon JP, Reixach N, Kelly JW. Sulfated glycosaminoglycans accelerate transthyretin amyloidogenesis by quaternary structural conversion. Biochemistry 2011;50:1001–1015. 21194234 10.1021/bi101822y PMC3035766

[205] Noborn F, O’Callaghan P, Hermansson E, Zhang X, Ancsin JB, Damas AM, Dacklin I, Presto J, Johansson J, Saraiva MJ, Lundgren E, Kisilevsky R, Westermark P, Li J-P. Heparan sulfate/heparin promotes transthyretin fibrillization through selective binding to a basic motif in the protein. Proc Natl Acad Sci U S A 2011;108:5584–5589. 21422279 10.1073/pnas.1101194108 PMC3078407

[206] Richards DB, Cookson LM, Berges AC, Barton SV, Lane T, Ritter JM, Fontana M, Moon JC, Pinzani M, Gillmore JD, Hawkins PN, Pepys MB. Therapeutic clearance of amyloid by antibodies to serum amyloid P component. N Engl J Med 2015;373:1106–1114. 26176329 10.1056/NEJMoa1504942

[207] Wisniewski T, Drummond E. APOE-amyloid interaction: therapeutic targets. Neurobiol Dis 2020;138:104784. 32027932 10.1016/j.nbd.2020.104784 PMC7118587

[208] Lewkowicz E, Jayaraman S, Gursky O. Protein amyloid cofactors: charged side-chain arrays meet their match? Trends Biochem Sci 2021;46:626–629. 34210544 10.1016/j.tibs.2021.05.003 PMC8415129

[209] Richards DB, Cookson LM, Barton SV, Liefaard L, Lane T, Hutt DF, Ritter JM, Fontana M, Moon JC, Gillmore JD, Wechalekar A, Hawkins PN, Pepys MB. Repeat doses of antibody to serum amyloid P component clear amyloid deposits in patients with systemic amyloidosis. Sci Transl Med 2018;10:eaan3128. 29298867 10.1126/scitranslmed.aan3128

